# Independent estimates of marine population connectivity are more concordant when accounting for uncertainties in larval origins

**DOI:** 10.1038/s41598-018-19833-w

**Published:** 2018-02-08

**Authors:** R. Nolasco, I. Gomes, L. Peteiro, R. Albuquerque, T. Luna, J. Dubert, S. E. Swearer, H. Queiroga

**Affiliations:** 1 Departamento de Física & CESAM - Centro de Estudos do Ambiente e do Mar, Universidade de Aveiro, 3810-193 Aveiro, Portugal; 2 Instituto de Investigacións Mariñas (CSIC), Eduardo Cabello 6, 36208 Vigo, Spain; 3 Departamento de Biologia & CESAM - Centro de Estudos do Ambiente e do Mar, Universidade de Aveiro, 3810-193 Aveiro, Portugal; 4 Mar. Biol. Research Group, Ghent University, 9000 Ghent, Belgium; 5 Coastal Ecology Research Group (EcoCost), Department of Ecology and Animal Biology, University of Vigo, Vigo, Spain; 6 School of BioSciences, University of Melbourne, Parkville, Victoria, 3010 Australia

## Abstract

Marine larval dispersal is a complex biophysical process that depends on the effects of species biology and oceanography, leading to logistical difficulties in estimating connectivity among populations of marine animals with biphasic life cycles. To address this challenge, the application of multiple methodological approaches has been advocated, in order to increase confidence in estimates of population connectivity. However, studies seldom account for sources of uncertainty associated with each method, which undermines a direct comparative approach. In the present study we explicitly account for the statistical uncertainty in observed connectivity matrices derived from elemental chemistry of larval mussel shells, and compare these to predictions from a biophysical model of dispersal. To do this we manipulate the observed connectivity matrix by applying different confidence levels to the assignment of recruits to source populations, while concurrently modelling the intrinsic misclassification rate of larvae to known sources. We demonstrate that the correlation between the observed and modelled matrices increases as the number of observed recruits classified as unknowns approximates the observed larval misclassification rate. Using this approach, we show that unprecedented levels of concordance in connectivity estimates (r = 0.96) can be achieved, and at spatial scales (20–40 km) that are ecologically relevant.

## Introduction

The majority of marine macroinvertebrates and fishes have a biphasic life cycle comprised of relatively sedentary benthic adults and potentially dispersive pelagic larvae. Benthic populations of these species exhibit some degree of connectedness, with the consequence that local recruitment may be decoupled from local larval production. This creates challenges for identifying the drivers of population replenishment and persistence, which are fundamental to our understanding of gene flow, adaptation and evolution in the sea^1^, and for proper fisheries management and biodiversity conservation^2,3^. Additionally, variability in ocean circulation on the time frame of larval life^4^ and the lack of knowledge on biological parameters that interact with the circulation and other characteristics of the physical-chemical environment mean that predictions on the extent and direction of marine larval dispersal cannot be derived from first principles. Because of this limitation, available reviews and syntheses^5–10^ advocate the use of multiple methods in order to increase confidence in empirical estimates of larval dispersal and population connectivity.

A variety of approaches have been applied to identify the origins and the destinations of pelagic marine larvae (ref.^5,6,8,11–14^ and literature therein), which fall into four main groups: visual tracking of marine larvae, artificial tags, natural tags, and numerical biophysical modelling. Visual tracking of individual larvae is the only direct method available, but can only be applied to large larvae with short Pelagic Larval Durations (PLDs) and thus has limited applicability. The remaining techniques have been extensively used, although many lack general applicability because they are dependent on particular life-history traits, physiology or anatomy of the target taxon or species. All techniques have intrinsic uncertainties that depend on type of markers, analytical procedures and statistical methodology. A matter of concern is how these internal uncertainties affect the comparison among dispersal estimates when multiple methods are used.

A literature review based on 507 research articles published since 1990 (see additional information in Supplementary Information 1-Literature review for definitions, a classification of methodologies and references) indicates that 41 studies^15–55^ have used at least two methodologies to estimate marine larval dispersal and connectivity matrices. The two most common approaches have been to use genetic markers and a numerical biophysical model, or the micro-chemistry of hard parts and a numerical biophysical model, but genetic markers and micro-chemistry, and combinations of genetic markers or micro-chemistry with current measurements, have also been employed. The review indicates that the degree of convergence between the different methods is widely taken as a measure of the trust that is put on the final solution: the more convergent the different methods, the higher the confidence on the description of the dispersal process. The majority of these assessments were qualitative, expressed as verbal descriptions of the patterns of dispersal that were obtained, with particular emphasis on the spatial coincidence of observed or predicted barriers to dispersal. A variety of methods were employed to produce semi-quantitative assessments (different approaches tested separately for significance, followed by numerical comparison of the test statistics) and quantitative assessments (a test statistics of the fit between the dispersal estimated by the different approaches was calculated and assessed), depending on the type of dispersal metrics that was employed: assessments of proportional variability explained by separate observed and predicted genetic isolation-by-distance^30^ or by separate isolation-by-geographic distance and isolation-by-oceanographic distance regressions^49,53,56^, Mantel tests between observed and/or predicted distance matrices^18,34,40,48,54^, log Bayes factors analysis that the predicted genetic structure fits the observed genetic structure^37^, sums of squared differences between predicted and observed allele frequencies^25^, multiple regression of genetic distance on oceanographic distance and environmental variables^28^, MANOVA of elemental ratios of individuals assigned to groups based on parentage^24^, and correlation between connectivity matrices^32^.

An important consideration on the use of empirical methods or models to infer dispersal and population connectivity is the confidence on the assignment to the population of origin. The empirical methods used by previous studies assign larvae or recruits to putative parental populations on a probabilistic fashion (based on assumptions of probability distributions of alleles or elements, number and size of populations, and other demographic processes), and have intrinsic uncertainties^57^. Three studies that did estimate a connectivity matrix based on genetics or elemental fingerprinting did explicitly incorporate this uncertainty into the decision of allocating larvae or recruits to parental populations, by specifying a posterior probability threshold for correct assignment (from 0.70 to 0.95^32,47,50^), while five studies simply allocated larvae or recruits to a given population when the posterior probability of pertaining to this population was higher than that of pertaining to any other population^15,19,30,44,52^. Numerical biophysical models also have intrinsic uncertainties associated with different biological and oceanographic causes^13,14^. Typically, the studies reviewed here provided some kind of temporal integration or used multiple runs with different environmental forcings, in order to smooth seasonal and inter-annual variability in currents. None of the studies provided information on sensitivity of the model to parameterization of sub-grid processes, nesting or resolution, although several of the studies were based on oceanographic models that have been extensively tested elsewhere (e. g.^17,26,31,34,35,39^). Most studies assumed fixed values for biological parameters, based on literature data, although a few used different biological scenarios in separate runs of the model.

Advancements on the merging of independent approaches to describe dispersal patterns have been to use connectivity matrices derived from biophysical models into population genetic models, in order to predict genetic structure. If the predicted genetic structure matches the observed structure, a case is made that migration mediated by oceanographic patterns of propagule transport influences gene flow. These studies used a derivation of the Bodmer & Cavalli-Sforza^58^ matrix model of migration to predict equilibrium allele frequencies after a variable number of generations^26,31,40,54,56^, or used modelled pairwise migration probabilities to inform a population model predicting allele frequencies at equilibrium^17,25,48^.

Most studies reviewed above used numerical biophysical models to obtain independent estimates of dispersal that could either be compared to empirical estimates, or that could feed population genetic models. None of the studies presented the models in a framework of model validation against observations, nor were they concerned with the uncertainty inherent to the empirical measurements of connectivity when comparing predictions of the models to empirical observations^59,60^. Only three studies explicitly accounted for uncertainty into the decision of allocating larvae or recruits to parental populations^32,47,50^, and only^32^ attempted a formal quantitative comparison between model predictions and observations. This uncertainty can be very large and probably depends on the number of populations. In^32^, which included 13 populations, 68% (262 in 382) individuals were discarded by applying a threshold level for correct assignment of 80%. In^47^, which considered only two populations, slightly less than 20% of the individuals were classified as unknowns, for a 0.95 probability of correct allocation.

Our review of the literature indicates that many of the studies did not use stringent rules to assign dispersers to their natal populations based on their probabilities of correct assignment, and when they did they did not investigate why these probabilities might vary, nor how the confidence level used would affect comparison among estimates. Thus, there is a clear need to explicitly address the challenges of comparing dispersal estimates across methods while addressing the issue of uncertainty in order to i) reduce this uncertainty wherever possible and ii) demonstrate that the convergent solution provides a robust estimate of the connectivity matrix.

In the present paper we addressed this issue in the Mediterranean mussel, *Mytilus galloprovincialis* Lamarck, using elemental fingerprinting and a numerical biophysical model. Our geographical domain is the west coast of the Iberian Peninsula. To do so we manipulated the observed (empirically-derived) connectivity matrix by applying different confidence levels to the assignment of recruits to the source populations. Recruits that failed to pass the prescribed confidence level were assigned to an unknown category. We manipulated the modelled connectivity matrix by using different population and larval biology scenarios. Moreover, we simulated the intrinsic variability of the geochemical signal by classifying modelled recruits as unknowns in a proportion equivalent to the misclassification rate of the larvae to their own sources, which is a measure of the inherent variability of the elemental profile. A second source of uncertainty was addressed by also classifying as unknowns the modelled recruits that originated outside the region for which elemental data was available. We demonstrate that the degree of convergence between the observed and modelled matrices increased as the proportion of recruits classified as unknowns approached the modelled proportion of unknowns, and that the increase in convergence is significantly different from that obtained with a random classification of recruits into an unknown origin.

## Methods

### Elemental fingerprinting and the generation of observed connectivity matrices

**Figure 1:**
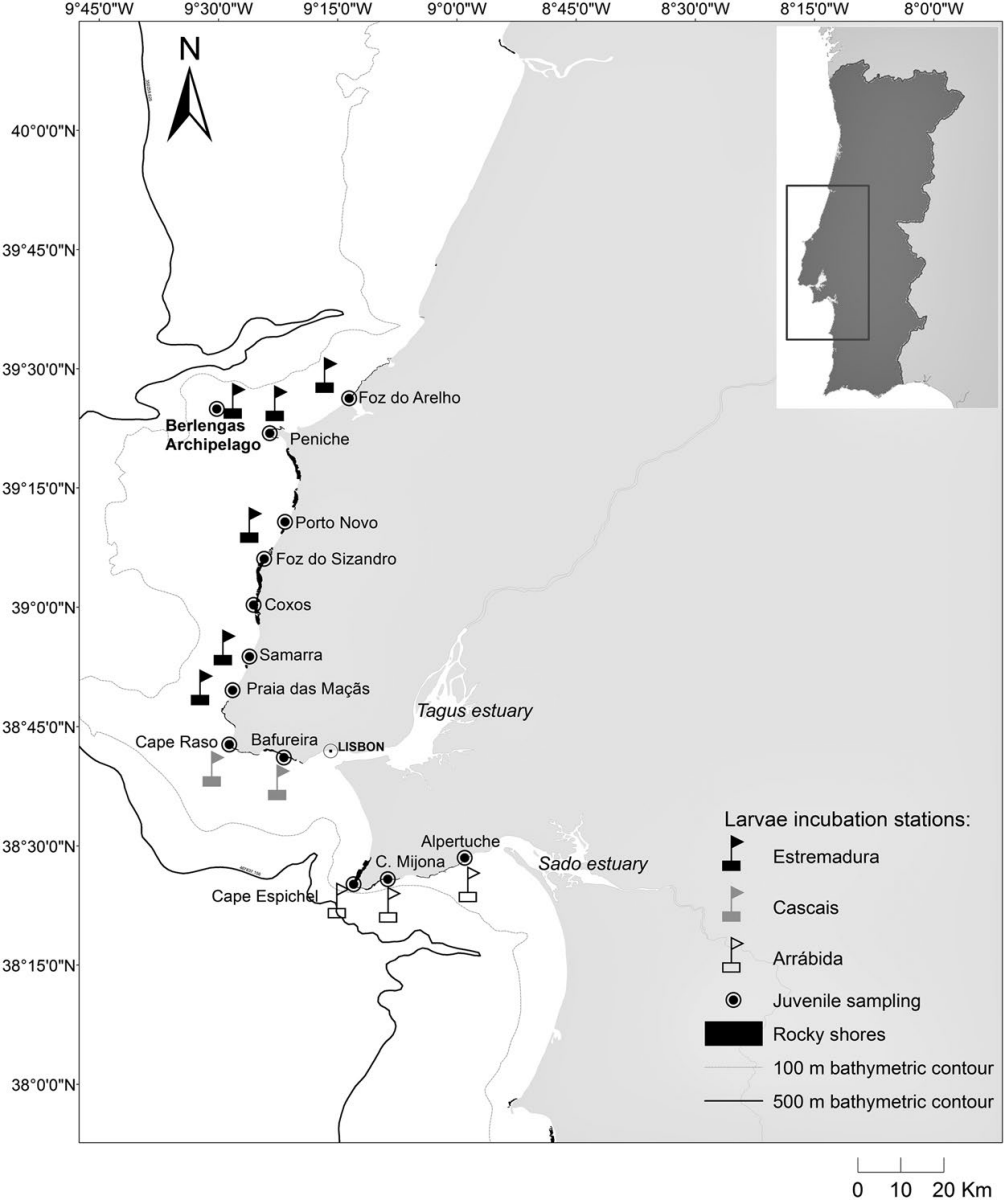
Map of larval incubation stations and juvenile sampling sites. Estremadura North: Berlengas, Peniche and Foz do Arelho. Estremadura South: Porto Novo, Samarra and Praia das Maçãs. Cascais Bay: Cabo Raso and Bafureira. Arrábida Bay: Cabo Espichel, Cova da Mijona and Alpertuche. For better visualization purposes, moorings in the map are illustrated more offshore than in the field (deployed at a depth of 15 to 20 m). Adapted from^61^.

The methodology used to obtain an atlas of geochemical natal signatures and for establishing the natal origin of the recruits is described in^61^. In brief, this methodology consisted of growing early laboratory-produced mussel embryos for 6 days inside incubators deployed in the field until a larval shell had clearly developed (70 to 140 μm shell length). Incubators were deployed at approximately 20 km intervals along the central coast of Portugal (Fig. 1), which is characterized by extensive rocky shores and is delimited by long stretches of almost continuous sandy shores to the north (150 km) and south (50 km). Six weeks after the start of the incubations, mussel juveniles were collected from rocky shores adjacent to each incubation site. Given the expected larval and juvenile growth rates at the temperature recorded during the study period (June-July of 2013), the time window of larval incubation should coincide with the period when the sampled recruits were produced. Larval shells and the larval portion of the recruits’ shells were then subjected to LA-ICPMS analysis using standard protocols (see^61^ for detailed methodology).

A jack-knifed linear Discriminant Function Analysis (DFA) of element-to-calcium ratios applied to the larval data produced a relatively low reclassification success at the site level (43.7% of cross-validated cases correctly classified), but a better discrimination at the region level (79.5%) when considering three regions: Estremadura (sites Berlengas, Peniche and Foz do Arelho, Porto Novo, Samarra and Praia das Maçãs), Cascais Bay (sites Cabo Raso, Bafureira) and Arrábida Bay (sites Cabo Espichel, Cova da Mijona, Alpertuche). An intermediate reclassification success (68.3%) was obtained when considering four regions, by splitting the large Estremadura region into two: Estremadura North (Berlengas, Peniche and Foz do Arelho) and Estremadura South (Porto Novo, Samarra and praia das Maçãs). A Monte-Carlo cross-validation technique^62^ indicated that randomly discarding up to 80% of the larvae did not have significant effects on the misclassification error relative to the full data set, confirming the capability to detect distinctive signatures for each region and sufficient sampling effort to account for variability within each region^62^.

The discriminant functions trained with the larval data were then used to assign recruits to natal origins, at the regional level, and to generate a series of observed connectivity matrices that differed depending on the confidence level applied during the assignment procedure. DFA assigns objects to previously defined groups based on the multivariate probability distribution of the dependent variables across objects within each group^63^. DFA calculates the posterior probabilities of each object belonging to each group and assigns an object to a specific group if the probability of pertaining to that group is higher than the probability of pertaining to the remaining groups, independently of the magnitude of probability differences. In the present case this introduces a source of uncertainty associated with the inter-individual variability of the elemental profile, which may result in incorrectly assigned recruits (Type 2 recruits; see below). Additionally, when assigning objects to groups DFA assumes that all objects belong to one of the *a priori* defined groups, and to none other. Our data set presumably violates this assumption because there is the possibility that recruits could have originated from outside the sampled region (Type 3 recruits; see below), although this should be minimized by the isolation of the sampled region by long stretches of coastline devoid of mussels. In order to account for these inherent types of uncertainty we used different confidence levels during the assignment procedure (Assignment Probability Thresholds, APT), based on the posterior probability thresholds of originating from the different populations: better-than-the-rest (none of the recruits classified as of unknown origin; recruits assigned to the population to which they have the better probability of belonging), 0.50, 0.75, 0.90, 0.95 and 0.99 (Table 1). These APT cover the range of confidence levels used in most practical applications and allowed us to test the sensitivity of the compliance between observed and modelled connectivity matrices to the confidence level used for generating the observed connectivity matrix.

**Table 1:** Definitions and codes of types of recruits, spawning regimes, larval behaviours, matrix spatial arrangements and assignment probability thresholds.

Types of recruits or Scenarios	Code
**Types of recruits**	
Recruits originating within the core region that are positively assigned to a specific origin.	Type 1
Recruits originated within the core region but of uncertain origin because of a natal signature not distinct enough to warrant a positive assignment to a specific origin.	Type 2
Recruits originated outside the core region and of unknown origin because of an unknown natal signature.	Type 3
**Spawning regimes**	
Continuous larval emission during each high tide until July 12.	S1
Continuous larval emission during each high tide until June 30; from that day on, discontinuous larval emission, skipping one of every two high tides until July 12.	S2
Continuous larval emission during each high tide until June 30; from that day on, discontinuous larval emission, skipping two of every three high tides, until July 12.	S3
Continuous larval emission during each high tide until July 1; from that day on, no more larvae were released.	S4
**Larval behaviours**	
Passive larvae.	Pa
Ontogenetic migration from a depth around 5 m until the pediveliger stage, followed by a migration to a depth around 12.5 m.	Om
Larvae dwelling in the bottom layer in shallow water and from 30 to 50 m in deeper water.	Bl
**Spatial arrangements**	
Origins: Estremadura, Cascais Bay and Arrábida Bay. Destinations: Estremadura, Cascais Bay and Arrábida Bay.	3 × 3
Origins: Estremadura, Cascais Bay and Arrábida Bay. Destinations: Estremadura North, Estremadura South, Cascais Bay and Arrábida Bay.	3 × 4
Origins: Estremadura North, Estremadura South, Cascais Bay and Arrábida Bay. Destinations: Estremadura North, Estremadura South, Cascais Bay and Arrábida Bay.	4 × 4
**Assignment Probability Thresholds**	
None of the recruits classified as of unknown origin; recruits assigned to the population to which they have the better probability of belonging.	Better-than-the-rest
Recruits classified as of unknown origin if the highest posterior probability of assignment was lower than the indicated value; otherwise, assigned to the population to which they have the better probability of belonging.	0.50, 0.75, 0.90, 0.99

### Biophysical numerical model and the generation of modelled connectivity matrices

The biophysical numerical model included two components: a nested oceanographic model based on the Regional Ocean Modelling System (ROMS), which produced velocity and temperature fields at 1 h intervals; and a biological model, implemented through a Lagrangian offline model that simulated the spatial and temporal distribution of mussel spawning, larval vertical migration behaviour, temperature-dependent planktonic larval duration and larval trajectories, based on the stored ROMS velocity and temperature fields interpolated at 300 s intervals. The nested model included a large domain extending from 12.5°W to 5.5°W and 34.4°N to 45.5°N (resolution of 1/27°; 60 vertical levels), which was used to provide boundary conditions to a medium domain corresponding to the West Iberian Margin (WIM; Cape St Vincent at 37°N to Cape Finisterre at 43°N, and from 11.5°W to the WIM coast at 8.5°W; resolution 1/60°; 45 levels). The medium domain was the target domain used for the dispersal simulations and was connected by two-way nesting to a small domain (from Figueira da Foz at 40.2°N to Sines at 37.8°N, extending to 10.5°W; resolution 1/180°, 45 levels), which encompassed the main region where natal and recruit signatures were collected (Fig. 1). A number of larvae proportional to the mussel biomass at each segment of the coast^64^ and to seasonal spawning activity^65^ was released adjacent to each rocky shore cell of the model and allowed to grow at a rate dependent on the thermal history predicted by ROMS, until a competent phase was reached^66,67^. If a larva found a rocky shore cell during the competent phase it was allowed to recruit; otherwise it would die. Because numerical models poorly resolve the coastal boundary layer where non-linear processes predominate^68^, a coastal buffer strip of 3 cells along the rocky shore was used as a settlement habitat. A more complete account of the biophysical model, environmental forcing and validation information based on^39,61,65–67,69–92^ can be found in the Supplementary Information 2-Biophysical model.

### Accounting for uncertainty: recruit origin and the construction of observed and modelled connectivity matrices

The Observed connectivity matrix refers to the geographical area for which natal and recruit elemental fingerprints were collected. The biophysical model covers a wider region, with additional origin and destination populations. Therefore, the Modelled connectivity matrix is larger than the Observed connectivity matrix. In the following description, whenever we refer to the core connectivity matrix(ces) we are referring to the area from where elemental fingerprints were sampled.

**Figure 2:**
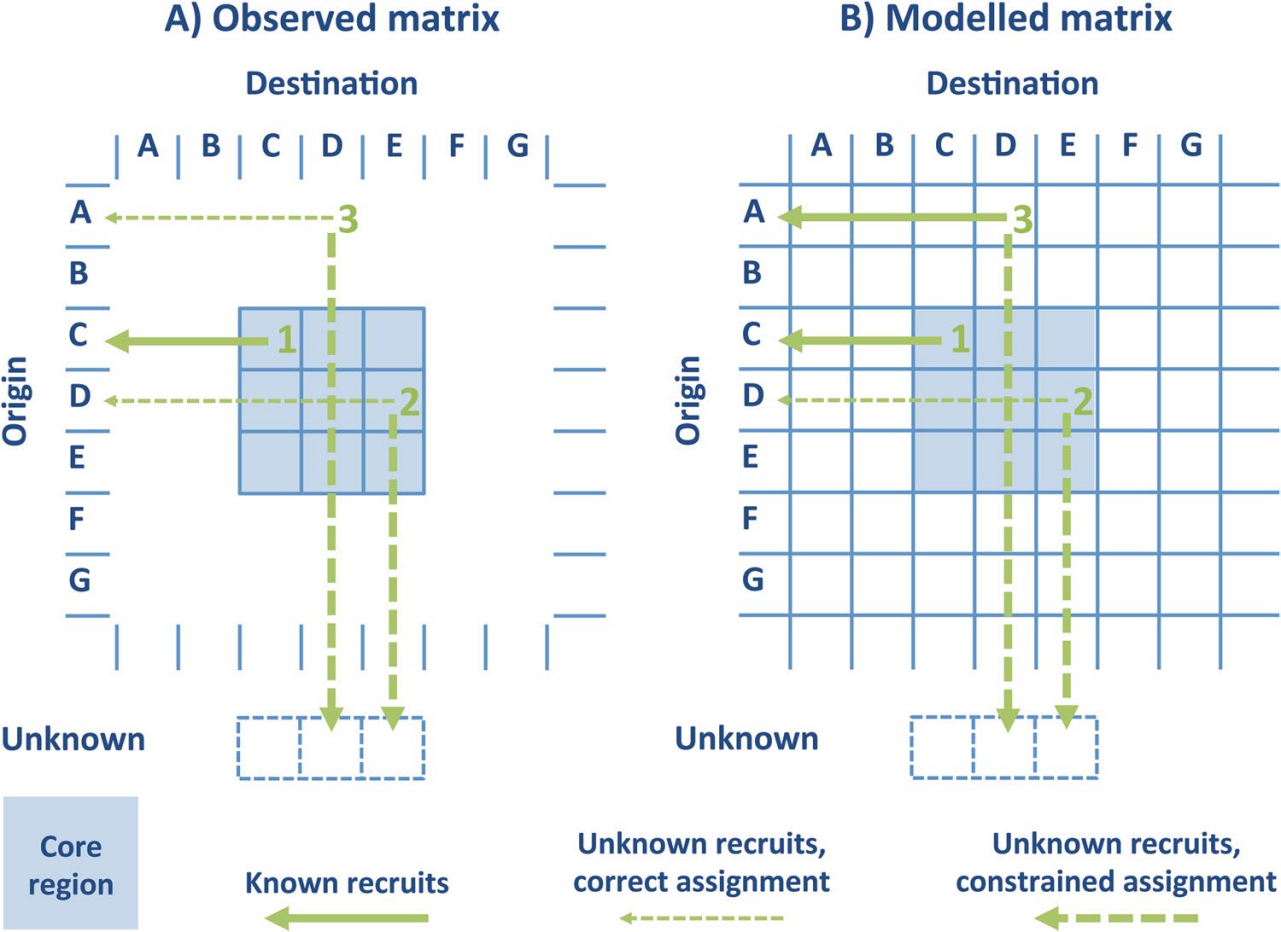
Observed (**A**) and Modelled (**B**) connectivity matrices for the 3 by 3 subdivision of the core region. The arrows illustrate the assignment of recruits into the populations of origin. Type 1 recruits (1): individuals recruited into the core region that originate within the core region and are assigned to origins within the core region. Type 2 recruits (2): individuals recruited into the core region that originate within the core region; assignment in the Observed matrix is not possible because of a poorly defined natal fingerprint and they are classified as unknowns; in the Modelled matrix they are classified as unknowns based on the probability of incorrect self-assignment of the larvae. Type 3 recruits (3): individuals recruited into the core regions that originate outside the core region; assignment in the Observed matrix is not possible because of an unregistered natal fingerprint and they are also classified as unknowns; in the Modelled matrix they are classified as unknowns to simulate the lack of knowledge about their natal signature.

When constructing the Observed connectivity matrix, the decision on the assignment of each recruit to a particular population of origin depends on the confidence level we wish to put in the assignment, i. e., depends on the selected posterior probability threshold of pertaining to that specific origin. With a higher confidence level we increase the number of unassigned recruits. In each particular case the unassigned individual has one of two possible origins: it may have originated within the core region but the elemental fingerprint of the origin is not distinct enough to warrant a positive assignment to the source population (Type 2 recruits in Fig. 2); or it may have originated from a population outside the core region (Type 3 recruits in Fig. 2). Type 2 recruits should be part of the connectivity core matrix but have to be assigned to an unknown origin. Type 3 recruits are not part of the core connectivity matrix because they originated outside the core region. They are also assigned to an unknown origin, because the natal signature of the population of origin is unknown. Type 1 recruits are those that are positively assigned to a specific origin population in the core matrix (see Fig. 2).

The Modelled, connectivity matrix is not affected by these sources of uncertainty because all recruits, irrespective of their origin and destination, can be “tracked back” by the model to their original populations (actually they are tracked forward from origin to destination or death). We simulated the uncertainty in the observations caused by the fact that the natal elemental signature is not distinctive enough to allow a positive assignment in all cases. To do this we assigned an unknown origin to a number of recruits into the core region that originated inside the core region, proportionally to the misclassification rate of the larvae. This forced some of the modelled recruits into Type 2 (see Fig. 2). All modelled recruits originating outside the core region but recruiting here are Type 3 recruits and not part of the connectivity matrix by definition (see Fig. 2). We also assigned these individuals to an unknown origin in order to simulate the lack of knowledge about their natal signature. Based on the observed elemental fingerprints a few of them would falsely be assigned to an origin within the core region because of an unclear natal fingerprint. This uncertainty cannot be simulated. We could predict the proportion of the modelled recruits that should falsely be classified into the core region based on the misclassification rate of the larvae (by assuming an average value of this rate for the whole area), but there is no way of predicting to which population of the core region these recruits should be assigned to. We assume this source of uncertainty is negligible because: i) the further away from the core area the likelier that the natal signatures differ from those of the core area, reducing the probability of falsely assigning these recruits to an origin inside the core area; and ii) there are long stretches of sandy shores to the north and south of the core area, effectively reducing the number of Type 3 recruits.

Given the above, we generated a series of Observed connectivity matrices that differed (see below) in the number of the partitions of the core region (3 different arrangements) and confidence level (6 levels). We also generated a series of Modelled connectivity matrices that differed (see below) in spawning regime (4 regimes), larval behaviour (3 behaviours) and partitioning of the core region (3 different arrangements). We corrected the core Modelled matrix for Type 2 recruits by subtracting from the predicted recruits in each cell a number proportional to the misclassification rate of the corresponding origin. Each row of the Modelled core matrix was therefore corrected by a different proportion. Modelled Type 2 and Type 3 recruits were included in an unknown row. Observed recruits that failed to pass the confidence level threshold were also included in an unknown row. In the above comparisons, Observed and Modelled matrices were standardized by dividing the number of recruits into each destination by the total number of recruits that settled into that destination, i. e., by the sum of the respective column. The rationale for this standardization is that the sampling of recruited individuals was constrained to an approximately constant number of individuals in each location, and did not reflect the distribution of settlement intensity among the sites. In contrast, the number of recruits predicted by the biophysical model did reflect the distribution of settlement intensity, because it incorporates not only the pattern of connectivity, but also the total number of larvae “hatched” in the model. That standardization allowed us to compare relative numbers of recruits into each destination originating from the different origins in both matrices.

### Accounting for uncertainty: mussel biology scenarios

In order to bracket the uncertainty regarding larval production and behaviour, we considered 4 scenarios of spawning regime and 3 scenarios of larval behaviour, and ran the biophysical model for all 12 combinations. The spawning regime scenarios attempted to simulate the reproductive exhaustion of individuals subsequent to the peak of gamete emission in spring/early summer described for the Iberian Peninsula, as described by^65,93^. Thus, the different regimes (Table 1) included constant larval spawning during high tide (mussels spawn only when submersed) along the entire rocky shore coast proportionally to population density during spring and early summer, followed by a progressive decline in larval emission towards the end of July, according to the following criteria: (S1) continuous larval emission during each high tide until July 12; (S2) continuous larval emission during each high tide until June 30; from that day on, discontinuous larval emission, skipping one of every two high tides until July 12; (S3) continuous larval emission during each high tide until June 30; from that day on, discontinuous larval emission, skipping two of every three high tides, until July 12; and (S4) Continuous larval emission during each high tide until July 1; from that day on, no more larvae were released. The larval behaviour scenarios (Table 1) included: (Pa) completely passive larvae, as implied by^81^; (Om) an ontogenetic migration from a depth around 5 m until the pediveliger stage, followed by a migration to a depth around 12.5 m, according to studies suggesting larvae tend to migrate deeper in the water column during development^77,82^; and (Bl) larvae dwelling in the bottom layer in shallow water and from 30 to 50 m in deeper water; this unrealistic scenario was intended to provide a contrast to the other two scenarios.

### Arrangement of the core matrix

We used 3 arrangements of the core connectivity matrix (Table 1) that were derived from *a priori* considerations about the oceanography and geometry of the region (which includes open coasts, capes, bays and coastal mountains), which can influence the probability of imprinting distinctive natal signatures^6,8^. The first was a 3 × 3 arrangement, with sampling sites for both origin and destination grouped into Estremadura, Cascais Bay and Arrábida Bay. This arrangement is based on the expectation of a distinct signature in the bays, caused by the influence of the Tagus and Sado rivers, and of a homogeneous signature along the more exposed Estremadura coast. In the second (3 × 4) and third (4 × 4) scenarios we kept the Cascais and the Arrábida bay regions, but made a distinction between the Estremadura North and South sections, separated by Cape Carvoeiro. This major cape induces strong and recurrent filament activities in response to upwelling events, which affect local oceanography and decouple to some degree both sections of the coast^86,94^. In the second scenario we expect a common natal signature for the whole Estremadura coast, but distinct settlement zones (Estremadura North and South) due to a two-cell circulation caused by the topographic influence of the cape. The third scenario considers the Estremadura North and South partition for both emission and settlement zones, based on the expectation of distinct natal signatures and circulation cells.

### Data availability

The original data consist of velocity, temperature and salinity fields predicted by the numerical model, occupying a total of ~340 GB of data. A version of these data fields with lower temporal (1 day) and spatial (~1/27°) resolution is publicly available through a THREDDS server at http://gmo.web.ua.pt/thredds/catalog/LD/2013/catalog.html.

## Results

### Generation of observed and modelled connectivity matrices

**Figure 3:**
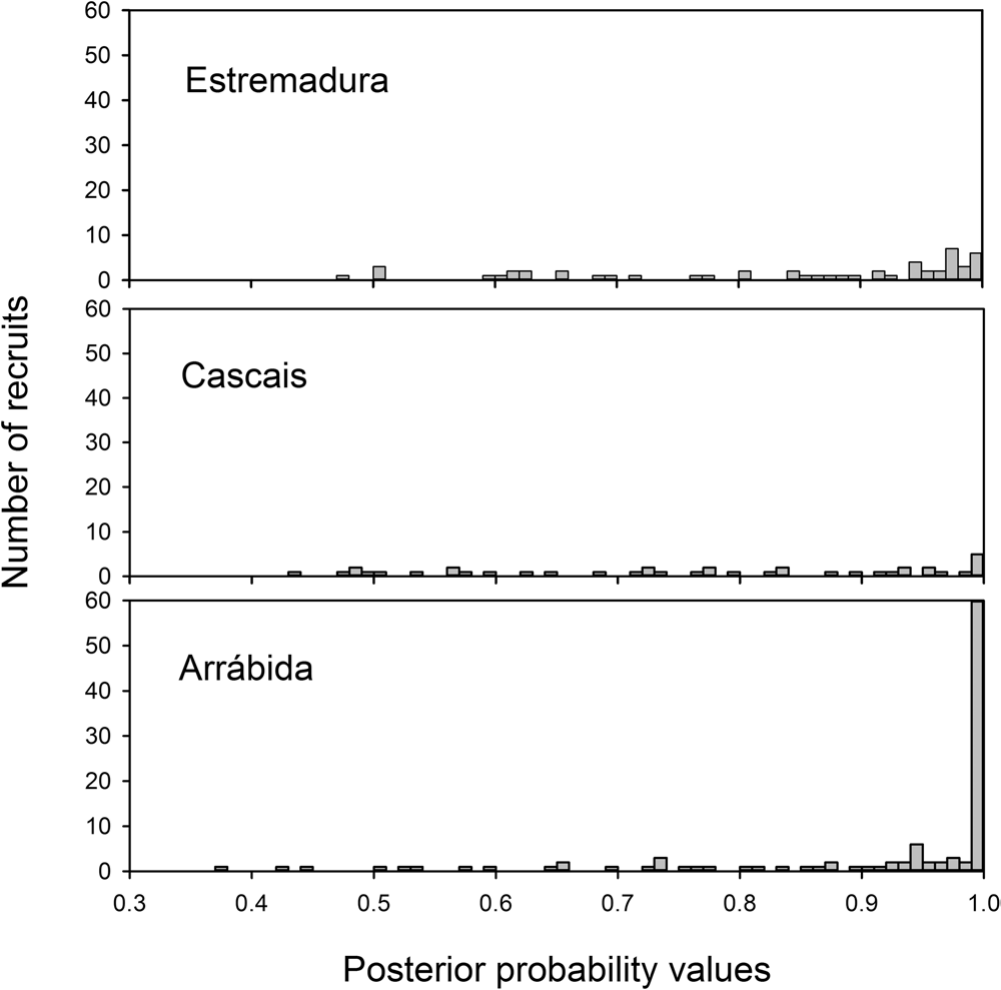
Posterior probabilities of assignment of mussel recruits into three putative origins, based on linear discriminant functions trained with larval shell elemental profiles.

**Figure 4:**
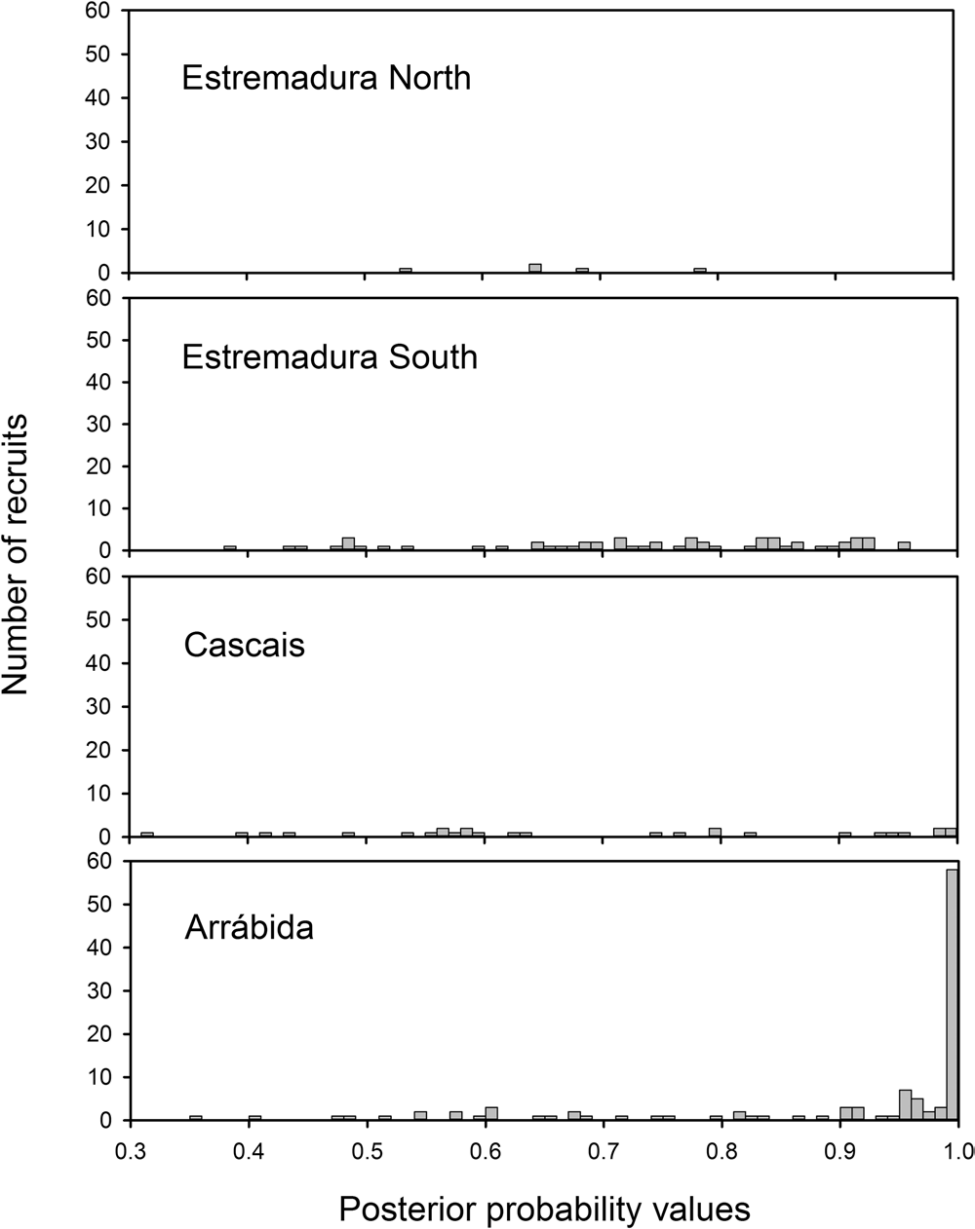
Posterior probabilities of assignment of mussel recruits into four putative origins, based on linear discriminant functions trained with larval shell elemental profiles.

The distributions of posterior probabilities of mussel recruits pertaining to each of the putative origins differed markedly among regions, for both the 3-region (Fig. 3) and 4-region (Fig. 4) connectivity matrices. In both cases Arrábida Bay was the most important source, with either 62 (APT - 0.99) or 82 (APT - 0.90) recruits originating from this region, when considering 3 regions, and either 61 (APT - 0.99) or 83 (APT - 0.90) recruits originating from this region, when considering 4 regions. In contrast, the number of recruits with assignment probabilities <0.90 was very similar among regions in the case of 3 regions (26, 26 and 25 for Arrábida, Cascais and Estremadura), but considerably more variable in the case of 4 regions (28, 20, 5 and 46 for Arrábida, Cascais, Estremadura North and Estremadura South). Thus, largely regardless of the method applied, Arrábida Bay was the main source of recruits to the different regions during the period of the study (see also^61^).

To compare connectivity matrices estimated by the two methods (geochemical fingerprint *vs* biophysical model), we based our analysis first on the Observed and Modelled connectivity matrices uncorrected for unknowns, and then on matrices corrected for both Type 2 and Type 3 recruits (Table 2). We did this because the elemental fingerprinting technique and the DFA cannot distinguish between the two sources of uncertainty, and therefore comparisons based on each correction separately are uninformative. However, we provide the full set of comparisons in the Supplementary Information 3-Matrix correlations.

**Table 2:** Pearson correlation coefficients between Observed and Modelled connectivity matrices for different combinations of larval behaviour, spawning regime, partitioning of the core region, and confidence level of the assignment of recruits into source populations. The top section refers to the core matrices without correction for unknowns; the bottom section refers to the core matrices plus unknown’s row, where the modelled matrix was corrected for Type 2 and Type 3 recruits simultaneously. Bold values indicate the highest correlation in each section.

CORE MATRICES (without unknown row); uncorrected modelled matrix		Larval behaviour/Spawning regime combinations											
		PaS1	PaS2	PaS3	PaS4	OmS1	OmS2	OmS3	OmS4	BlS1	BlS2	BlS3	BlS4
Partitioning of the core region/Confidence level combinations	3 × 3% 99	0.64	0.72	0.77	0.87	0.58	0.67	0.75	0.82	0.28	0.31	0.34	0.38
	3 × 3% 95	0.77	0.84	0.88	0.94	0.71	0.79	0.86	0.90	0.41	0.44	0.47	0.51
	3 × 3% 90	0.77	0.84	0.88	0.94	0.72	0.80	0.86	0.91	0.41	0.44	0.47	0.50
	3 × 3% 75	0.80	0.86	0.90	**0.96**	0.74	0.82	0.88	0.92	0.45	0.49	0.52	0.55
	3 × 3% 50	0.79	0.85	0.89	0.94	0.72	0.80	0.87	0.91	0.41	0.44	0.47	0.51
	3 × 3 Better	0.77	0.83	0.88	0.93	0.70	0.78	0.85	0.89	0.38	0.42	0.45	0.49
	3 × 4% 99	0.41	0.48	0.53	0.63	0.40	0.49	0.56	0.61	0.15	0.17	0.19	0.22
	3 × 4% 95	0.61	0.67	0.71	0.79	0.59	0.67	0.73	0.78	0.33	0.36	0.38	0.41
	3 × 4% 90	0.62	0.69	0.72	0.80	0.60	0.68	0.75	0.79	0.34	0.37	0.39	0.41
	3 × 4% 75	0.62	0.68	0.72	0.79	0.61	0.69	0.74	0.77	0.37	0.39	0.42	0.44
	3 × 4% 50	0.59	0.65	0.69	0.75	0.58	0.66	0.72	0.74	0.32	0.35	0.37	0.40
	3 × 4 Better	0.56	0.62	0.66	0.72	0.55	0.63	0.69	0.71	0.29	0.31	0.34	0.36
	4 × 4% 99	0.40	0.47	0.50	0.60	0.38	0.46	0.52	0.56	0.13	0.15	0.17	0.19
	4 × 4% 95	0.42	0.49	0.53	0.61	0.41	0.49	0.55	0.58	0.16	0.18	0.20	0.22
	4 × 4% 90	0.52	0.58	0.61	0.68	0.51	0.57	0.62	0.63	0.25	0.26	0.29	0.29
	4 × 4% 75	0.63	0.68	0.71	0.76	0.61	0.66	0.70	0.68	0.35	0.36	0.38	0.37
	4 × 4% 50	0.66	0.71	0.73	0.78	0.64	0.69	0.73	0.70	0.38	0.38	0.41	0.40
	4 × 4 Better	0.65	0.70	0.72	0.76	0.64	0.69	0.72	0.69	0.37	0.38	0.40	0.38
CORE MATRICES + UNKNOWNS; modelled matrix corrected for Type 2 and Type 3 recruits		Larval behaviour/Spawning regime combinations											
		PaS1	PaS2	PaS3	PaS4	OmS1	OmS2	OmS3	OmS4	BlS1	BlS2	BlS3	BlS4
Partitioning of the core region/Confidence level combinations	3 × 3% 99	0.47	0.55	0.61	0.76	0.51	0.58	0.62	0.71	0.07	0.09	0.11	0.14
	3 × 3% 95	0.63	0.71	0.77	0.88	0.65	0.72	0.76	0.83	0.20	0.23	0.25	0.28
	3 × 3% 90	0.69	0.77	0.83	0.93	0.71	0.78	0.83	0.88	0.26	0.29	0.32	0.35
	3 × 3% 75	0.70	0.77	0.82	0.88	0.70	0.77	0.82	0.83	0.36	0.39	0.42	0.45
	3 × 3% 50	0.57	0.63	0.66	0.66	0.55	0.61	0.65	0.63	0.35	0.37	0.39	0.41
	3 × 3 Better	0.53	0.58	0.62	0.61	0.50	0.56	0.60	0.57	0.33	0.35	0.38	0.40
	3 × 4% 99	0.55	0.60	0.63	0.73	0.52	0.57	0.61	0.67	−0.06	−0.04	−0.03	−0.02
	3 × 4% 95	0.63	0.70	0.74	0.83	0.60	0.66	0.71	0.76	0.08	0.10	0.11	0.13
	3 × 4% 90	0.63	0.70	0.74	0.83	0.59	0.66	0.72	0.78	0.16	0.18	0.20	0.22
	3 × 4% 75	0.56	0.63	0.67	0.73	0.54	0.61	0.67	0.70	0.28	0.30	0.31	0.33
	3 × 4% 50	0.32	0.37	0.39	0.42	0.31	0.36	0.41	0.42	0.30	0.31	0.33	0.34
	3 × 4 Better	0.25	0.30	0.32	0.35	0.24	0.30	0.34	0.35	0.28	0.29	0.31	0.32
	4 × 4% 99	0.89	0.90	0.90	0.92	0.84	0.85	0.86	0.86	0.47	0.46	0.48	0.43
	4 × 4% 95	0.89	0.92	0.93	**0**.**96**	0.86	0.88	0.90	0.92	0.48	0.47	0.49	0.45
	4 × 4% 90	0.86	0.90	0.91	0.95	0.83	0.86	0.89	0.91	0.47	0.46	0.49	0.45
	4 × 4% 75	0.75	0.79	0.82	0.86	0.73	0.78	0.81	0.83	0.45	0.44	0.47	0.43
	4 × 4% 50	0.33	0.38	0.41	0.46	0.32	0.38	0.43	0.45	0.22	0.23	0.25	0.25
	4 × 4 Better	0.21	0.26	0.29	0.34	0.21	0.27	0.32	0.34	0.14	0.15	0.17	0.17

Pa = passive larvae. Om = larvae migrating ontogenetically. Bl = larvae dwelling in the bottom layer. S1 = continuous larval emission during each high tide until July 12. S2 = continuous larval emission during each high tide until June 30, then larval emission skipping one of every two high tides until July 12. S3 = continuous larval emission during each high tide until June 30, then larval emission skipping two of every three high tides until July 12. S4 = Continuous larval emission during each high tide until July 1, no more larvae released afterwards. 3 × 3, 3 × 4 and 4 × 4 = spatial organization of the core region into 3 or 4 origin x destination cells. Better = recruits assigned into an origin when the probability of pertaining to that origin is better that that of pertaining to any other origin. %99, %95, %90, %75, %50 = recruits assigned into an origin when the probability of pertaining to that origin is larger that the level indicated.

From the set of comparisons without correcting for unknowns, the best correlations correspond to the 3 × 3 spatial grids, reaching correlation coefficients over 0.90 for several scenarios of spawning and larval behaviour (Table 2). However, when larvae were forced to dwell in the bottom layer (Bl) the correlations decreased dramatically (to an average of 0.44 correlation). The 3 × 3 spatial grid scenarios that incorporated passive (Pa) or ontogenetic behaviours (Om), and simulated reproductive exhaustion (progressive decline in larval emission towards the end of the study period, S3 and S4), produced high correlation coefficients between the two matrices. This was particularly true (average 0.93 correlation) when no larvae were released from July onwards (S4). Using spatial grids with higher spatial resolution (3 × 4 and 4 × 4 matrixes) caused the correlations to drop progressively, although they were still elevated (r > 0.70) in some scenarios. This reduction is related to a decrease in accuracy of recruit assignment based on shell geochemistry, as the signatures are not distinct at this spatial resolution (DFA reclassification success for the larvae in^61^). In both 3 × 3 and 4 × 4 arrangements, recruits predicted by the biophysical model to settle in the Estremadura (north and south) region showed the worst fit to the observations, but the model was well fitted to describe natal origins for recruits which settle in the Arrábida Bay, and to a lesser degree in the Cascais Bay (Supplementary Information 4-Matrix adjustment). When we changed the APT (Table 2), we obtained a similar pattern for most combinations, where best model fits correspond to thresholds around 0.75–0.95. The less restrictive scenario (APT better-than-the-rest and 0.50) showed the lowest correlation between Observed and Modelled matrices, with the exception of the 4 × 4 core matrices scenarios. For APTs of 0.75 to 0.99, correlations were quite similar for most of the scenarios, and maxima often fell around 0.90. That seems to indicate that the model reproduces the observed data when we maintain a moderate to high threshold, except for the 4 × 4 scenarios where the uncertainty of the geochemical data is higher.

If we now take into consideration the recruits of unknown origin (Type 2 and Type 3, i.e., all the ones that failed to be successfully classified to one of the possible origins; Table 2) a different picture emerges. The contrasts between larval behaviours and spawning regimes still followed the same patterns as in the preceding case, but now the effect of increasing spatial resolution differs. When we corrected for Type 2 and Type 3 (Table 2) the correlations increased considerably in the higher spatial resolution scenarios. This effect is related to the increase in the number of unknowns in the geochemical classification with increasing spatial resolution, resulting in an improved fit between the observed and predicted recruits in the 4 × 4 grid, especially in the Estremadura region (Supplementary Information 4-Matrix adjustment). It is interesting to note that the biophysical model very accurately described natal origins for the Arrábida recruits as well, followed by those that recruited into Cascais. Again, we observed the same pattern as before, with higher correlations with APTs between 0.75 and 0.95 (Table 2).

### Assessing the causes of convergence between observed and modelled connectivity matrices

Independently of whether we consider only the core connectivity matrices, or the connectivity matrices with an unknown row (origin), increasing the APTs increased matrix correlations up to a point between 0.75 and 0.95, after which matrix correlations decreased again (Table 2). Given this pattern, we make two predictions. The first prediction is that this effect is different from a random deletion of recruits from the Observed matrix. Increasing the confidence level from the “better-than-the-rest” case is akin to removing outliers from the Observed matrix, so we should expect that removing recruits at random would not result in an increased correlation. On the other hand, by being too strict in the assignment of recruits we could be removing individuals from the Observed matrix that are correctly classified, resulting in a decreased correlation. The second prediction is that the number of excluded recruits that provides the best correlation should logically match the number of those with a poorly defined elemental signature, plus those that originate from outside the core region. The first case reflects the compounded effect of assigning recruits to an unknown origin based on the misclassification rate of the larvae into their source population (i.e., the proportion of larvae incorrectly self-assigned in each region), which is a measure of the inherent variability of the elemental signature. That number is the number of Type 2 recruits that are assigned to the unknown row in the Modelled connectivity matrix. The second case is the number of Type 3 recruits.

We tested both predictions only for the core connectivity matrices, and for the connectivity matrices with an unknown row composed of Type 2 and Type 3 recruits, for the combination of continuous larval emission during each high tide until July 1 (S4) and passive larvae (Pa), which were the best biological scenarios overall, and for all spatial arrangements of the core matrix (3 × 3, 3 × 4 and 4 × 4). We used a bootstrap approach in order to test the first prediction. For each APT (0.50, 0.75, 0.90, 0.95 and 0.99) we generated 1000 Observed connectivity matrices by randomly discarding, from the better-than-the-rest matrix, a number of recruits equal to the sum of the worst classified recruits into every source. Each of the 1000 randomly adjusted Observed matrices for a given confidence level was then correlated with the corresponding Modelled matrix, and the frequency distribution of the correlation coefficients was generated. The correlation coefficient obtained from the comparison between the Observed matrix correctly adjusted for the confidence level and the Modelled matrix was then compared to that frequency distribution. To test the second prediction we calculated the difference between the proportion of recruits classified as unknowns in the Observed matrix for each confidence level and the proportion of modelled unknowns, and plotted the correlation coefficient against this quantity.

**Figure 5:**
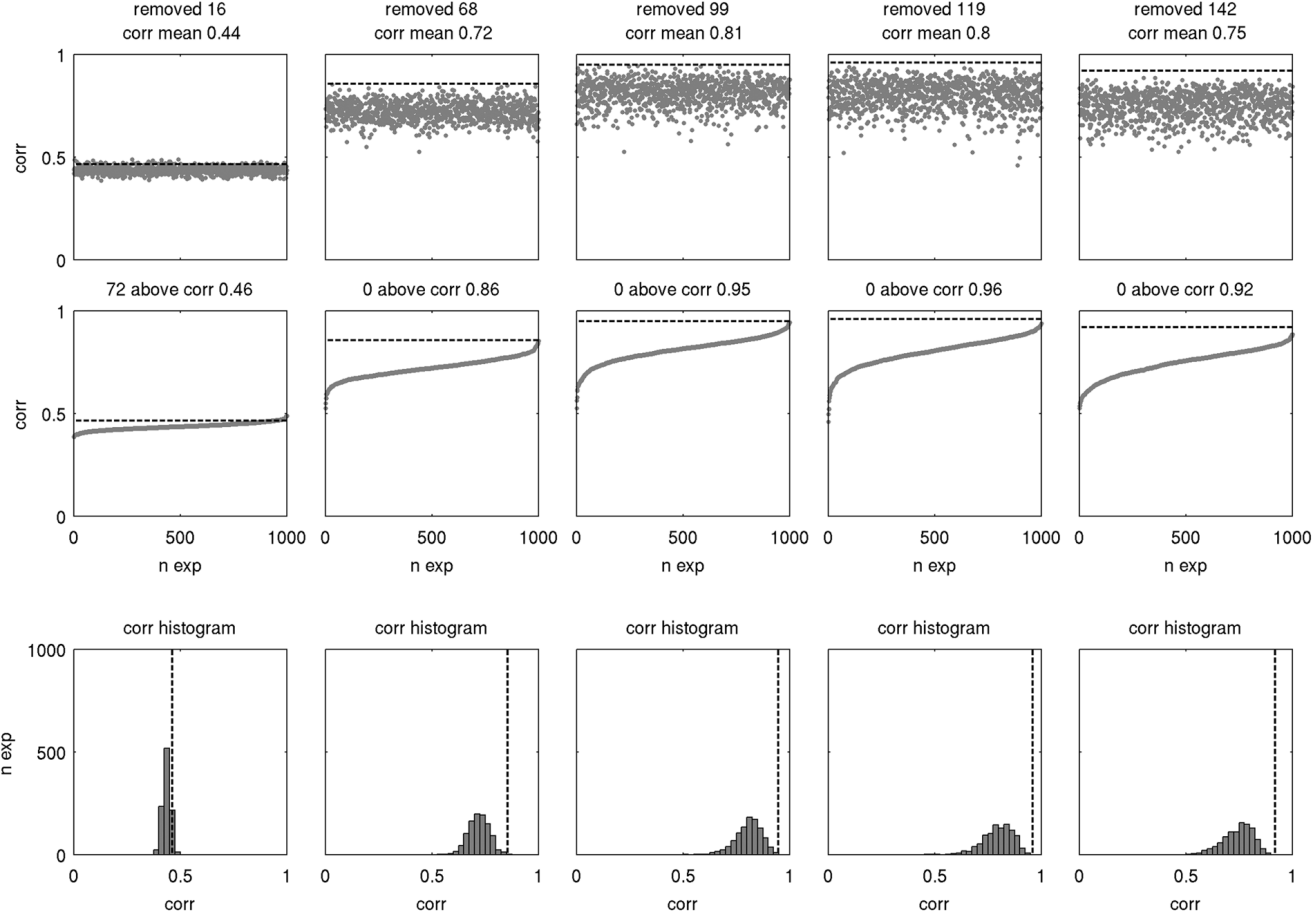
Effect on the matrix correlation coefficient of randomly excluding from the observed matrix a number of individuals equal to the number of observed individuals correctly classified as unknowns for each confidence level (columns are Assignment Probability Thresholds (APTs) of 0.50, 0.75, 0.90, 0.95 and 0.99), based on 1000 trials for each threshold. In each graph, the dashed line indicates the correlation coefficient that was obtained by removing those recruits that correctly failed to pass the APT. First row: distribution of correlation coefficients by trial number; the number of removed individuals is indicated above each graph. Second row: the same, but correlation coefficients ranked by value; the number of trials with a correlation coefficient above that obtained by removing those recruits that correctly failed to pass the posterior probability threshold is indicated above each graph. Third row: frequency distribution of the correlation coefficients. Removing the recruits that correctly failed to pass the APT resulted in a correlation coefficient significantly higher than that obtained by a random deletion of recruits at p < 0.0001 (****). “corr” = correlation coefficient. The figure only shows results for the 4 × 4 arrangement, passive larvae and the S4 spawning scenario (see Supplementary Information 5-Prediction 1 for other scenarios).

**Figure 6:**
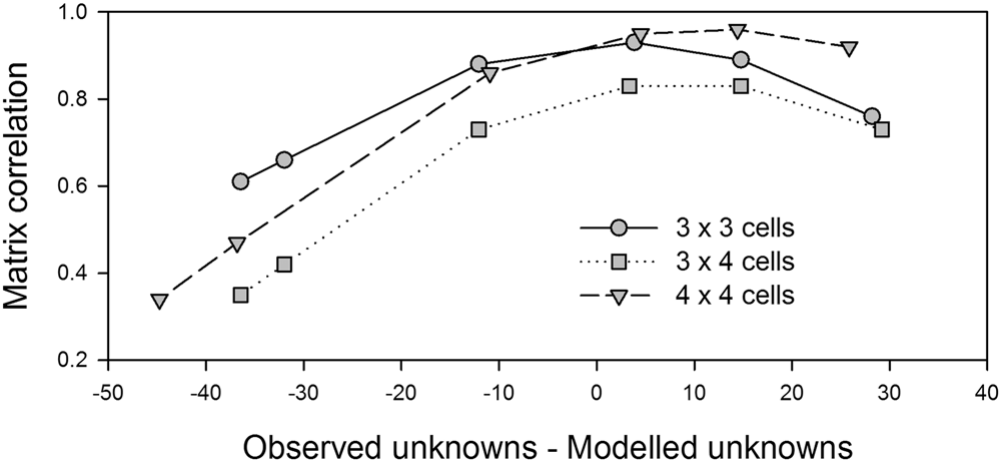
Relationship between the matrix correlation coefficient and the difference between the numbers of observed and modelled recruits classified as unknowns, for three different arrangements of the connectivity matrices. The number of observed recruits classified as unknowns changes with the threshold level (from left to right, APTs better-than-the-rest, then above 0.50, 0.75, 0.90, 0.95 and 0.99). The number of modelled recruits classified as unknowns depends on the misclassification rate of the larvae into their source population (proportion of larvae incorrectly self-assigned in each region; Type 2 recruits) and on those that originate from outside the core region (Type 3 recruits). The figure only shows results for passive larvae and the S4 spawning scenario (see Supplementary Information 6-Prediction 2 for other scenarios).

In 19 cases out of 30 comparisons, the improvement of the matrix correlation obtained by increasing the APT was significantly different from that obtained by a random deletion of recruits from the Observed matrix (Supplementary Information 5-Prediction 1). The cases where the improvement was most significant corresponded to the 4 × 4 spatial arrangement (Fig. 5, p < 0.0001 for the 0.75, 0.90, 0.95 and 0.99 APTs), which included also the highest correlation coefficient obtained across all scenarios (r = 0.96, Table 2, passive larvae (Pa), cessation of spawning after July (S4), 0.95 APT, 4 × 4 spatial arrangement). In the case of the 3 × 3 and 3 × 4 spatial arrangements the matrix correlation peaked when the difference between observed and modelled unknown recruits approached zero (at an APT of 0.90), and at a slightly positive value in the case of the 4 × 4 arrangement (at an APT of 0.95; Fig. 6).

Visual inspection of Observed and Modelled connectivity matrices for the 0.95 APT (Table 3; other thresholds not shown, but very similar results were obtained for 0.90; see also Supplementary Information 6-Prediction 2 for Type 2 and Type 3 recruits) indicates that the poorest performance of the model relative to the observations occurred in the Estremadura region. This is especially evident in the case of the 4 × 4 arrangement, where observations indicate supply to Estremadura S and Estremadura N from the southern regions, while the model indicates higher retention or supply from the north, especially in Estremadura N. Both observations and model results concur in identifying the Arrábida Bay as a region of high retention but also as a major supplier to Cascais and Estremadura S.

**Table 3:** Observed and Modelled connectivity matrices obtained for the scenarios of passive larvae and cessation of spawning after July, for the 3 × 3, 3 × 4 and 4 × 4 spatial arrangements. The top six panels refer to the core matrices without correction for unknowns and the better-than-the-rest assignment probability threshold; the bottom six panels refer to the core matrices plus unknown’s row for a 0.95 assignment probability threshold, where the Modelled matrix was corrected for Type 2 and Type 3 recruits simultaneously.

**Observed**, **better-than-the-rest case**					**Modelled**, **without correction for Type 2 or Type 3 recruits**				
Destination Origin	Estremadura	Cascais	Arrábida		Destination Origin	Estremadura	Cascais	Arrábida	
**Core connectivity matrices (without correction for unknowns)**									
Estremadura	34	17	9		Estremadura	58	9	0	
Cascais	24	7	16		Cascais	5	13	1	
Arrábida	41	77	74		Arrábida	37	78	99	
**Destination Origin**	**Estremadura N**	**Estremadura S**	**Cascais**	**Arrábida**	**Destination Origin**	**Estremadura N**	**Estremadura S**	**Cascais**	**Arrábida**
Estremadura	33	35	17	7	Estremadura	100	52	9	0
Cascais	43	7	7	19	Cascais	0	6	13	1
Arrábida	23	57	77	74	Arrábida	0	42	78	99
**Destination Origin**	**Estremadura N**	**Estremadura S**	**Cascais**	**Arrábida**	**Destination Origin**	**Estremadura N**	**Estremadura S**	**Cascais**	**Arrábida**
Estremadura N	7	1	0	0	Estremadura N	67	4	0	0
Estremadura S	37	35	17	14	Estremadura S	33	48	9	0
Cascais	33	4	3	9	Cascais	0	6	13	1
Arrábida	23	59	80	77	Arrábida	0	42	78	99
**Observed**, **0**.**95 assignment probability threshold**					**Modelled**, **corrected for Type 2 and Type 3 recruits**				
**Destination Origin**	**Estremadura**	**Cascais**	**Arrábida**		**Destination Origin**	**Estremadura**	**Cascais**	**Arrábida**	
**Connectivity matrices with unknown row**									
Estremadura	14	7	0		Estremadura	39	8	0	
Cascais	6	0	2		Cascais	2	8	0	
Arrábida	23	63	49		Arrábida	21	59	61	
Unknown	57	30	49		Unknown	38	25	39	
**Destination Origin**	**Estremadura N**	**Estremadura S**	**Cascais**	**Arrábida**	**Destination Origin**	**Estremadura N**	**Estremadura S**	**Cascais**	**Arrábida**
Estremadura	17	12	7	0	Estremadura	22	47	8	0
Cascais	13	0	0	2	Cascais	0	4	8	0
Arrábida	5	38	63	49	Arrábida	0	31	59	61
Unknown	65	50	30	49	Unknown	78	18	25	39
**Destination Origin**	**Estremadura N**	**Estremadura S**	**Cascais**	**Arrábida**	**Destination Origin**	**Estremadura N**	**Estremadura S**	**Cascais**	**Arrábida**
Estremadura N	0	0	0	0	Estremadura N	13	4	0	0
Estremadura S	0	3	0	0	Estremadura S	3	19	4	0
Cascais	8	0	0	0	Cascais	0	4	8	0
Arrábida	7	43	67	51	Arrábida	0	31	59	61
Unknown	85	54	33	49	Unknown	84	42	30	39

## Discussion

### Comparison between observed and modelled connectivity matrices

In the present study we manipulated an Observed connectivity matrix, derived from geochemical information of mussel larval and recruit shells, by applying different assignment probability thresholds (APTs) to the classification of recruits into the source populations based on the posterior probabilities of assignment. Recruits that failed to pass the prescribed APT were assigned to an unknown category. We also manipulated a Modelled connectivity matrix derived from a biophysical model by using different population and larval biology scenarios. Moreover, we simulated the intrinsic variability of the geochemical signal by classifying modelled recruits as unknowns in a proportion equivalent to the misclassification rate of the larvae to their own sources, which is a measure of the inherent variability of the elemental profile. A second source of uncertainty was addressed by also classifying as unknowns the modelled recruits that originated outside the region for which elemental data were available. We obtained a very good convergence between the two methods at the lowest spatial resolution when no correction for unknowns was applied, with correlation coefficients *r* up to 0.96, but a worse fit at the highest spatial resolution with r < 0.76. When we corrected for unknowns the convergence between the two methods at the higher spatial resolution increased substantially to values of r > 0.80 and up to 0.93 and 0.96, for APTs between 0.90 and 0.95, passive or ontogenetically migrating larvae, and realistic spawning scenarios. As far as we know, there is no precedent for this level of convergence between two independent estimates of larval dispersal and connectivity at spatial scales below 40 km.

The interpretation of the fit between the two approaches requires a phenomenological interpretation of the dispersal process captured during this event^61^. The geochemical signatures indicated an overall northward dispersal of larvae, with those originating in the Arrábida Bay contributing disproportionally to the Cascais Bay and the Estremadura regions. This northward dispersal event runs contrary to the average circulation along the Portuguese coast during spring and summer, associated with upwelling circulation^95^, but is consistent with concurrent wind data that shows a 3-week long upwelling relaxation episode that took place just prior to the sampling of the recruits^61^. The relaxation episode was accompanied by a distinct temperature increase caused by the northward advection of warm waters, which was well captured by the biophysical model (Supplementary Information 2-Biophysical model). The high correlation coefficients obtained with a 3 × 3 spatial arrangement of the core zone, with passive and ontogenetic larval behaviour scenarios, are a consequence of the small spatial resolution overall (about 20, 30 and 70 km in the Cascais, Arrábida and Estremadura regions, respectively). As we increased spatial resolution by subdividing the Estremadura region we decreased the ability to assign recruits to their source populations based on the natal signatures, as the spatial resolution fails to be adequate to achieve good geospatial distinct chemical signals. However, when we incorporated the unknowns into a virtual box, both in the Observed and in the Modelled matrices, there still was a high correlation coefficient (r > 0.80) for a large range of biological scenarios and APTs, reaching a maximum of 0.96. That is, by explicitly modelling the uncertainty sources of the elemental fingerprinting technique, we were able to simultaneously increase the overall spatial resolution of the analysis (20, 30, 40, 30 km, for the Cascais, Arrábida, Estremadura south and Estremadura north regions, respectively) and the fit of the model to the observations.

### Assessing the causes of convergence between observed and modelled connectivity matrices

The numerical changes in the correlation coefficient with the shifting APTs were not due to random effects, with maximum correlations occurring when number of observed unknowns approached modelled unknowns, or slightly exceeded them in the case of the 4 × 4 spatial scenario. This last result suggests that the model underestimates the contribution of Type 3 recruits, or that the correction for Type 2 recruits has been overestimated, which could result from a small sample of the posterior probabilities as spatial resolution was increased. Other discrepancies between the observations and the model were the poor match in the Estremadura region. These discrepancies may arise from limitations of the elemental fingerprinting technique and of the model. Elemental fingerprinting requires that sufficient chemical variability of the water be present over space, but also that the chemistry of the calcified structures in some way reflects the physicochemical properties of the water. Controlled laboratory experiments indicate linear relationships between the concentrations of several elements in seawater and in calcified structures (mollusc larval shells and statoliths^96,97^, but also interactive effects of temperature and salinity (fish otoliths^98^; mollusc larval shells Andreia Carvalho & Laura Peteiro, unpublished data) that will influence the multivariate distribution of elements in the target structure and may complicate the probabilistic assignment of individuals and the interpretation of the patterns. The biophysical model on the other hand is constrained by its internal variability and may not be resolving appropriately all details of the oceanography and biology. For instance, although the model configuration is designed to solve the continental shelf circulation at the scale of the Western Iberian Margin, the inner continental shelf circulation is influenced by local cross-shelf winds and surface gravity waves (not solved), and is characterized by a logarithmic shoreward decrease in current velocity at scales of 1–2 km^68^, which likely affect the estimates of along-shore transport. Additionally, although we have obtained consistent estimates of dispersal across a range of spawning and of larval behaviour scenarios (except in the case of unrealistic bottom-dwelling larvae^77,81^), we used growth and mortality rates derived from the literature^66,67^ without a formal assessment of the model sensitivity to their variability.

### Future directions

In the present study we obtained high correlations (r = 0.96) between Observed and Modeleld connectivity matrices obtained by both approaches at a high spatial resolution (20–40 km), after discarding all recruits that failed to pass a stringent assignment probability threshold (APT = 0.95), in spite of other internal sources of error inherent to either methodology. Most of these recruits originated from the Arrábida Bay, which is distinguished from the other sources by a well-defined elemental signature. An argument can be drawn that, if the model describes these larvae with high certainty, it should also be well fitted to predict mussel larvae dispersal and trajectories in the remaining central Portuguese west coast. We propose that targeting dispersing individuals for which we have of high certainty of assignment to a natal population is an effective way of validating biophysical models of larval dispersal, allowing stronger inferences on population connectivity relevant for the management of marine populations. Presently, the demonstration of the biophysical model accuracy at smaller spatial scales seems to be limited by the resolution of the geochemical fingerprinting technique. The approach taken here also highlights the potential in using these two techniques in an integrated manner, in order to compensate for, and explore, different spatial resolutions and sources of uncertainty^5–10,13^, and opens the door to effectively combine the two techniques to investigate the ability of biophysical models *per se* to describe a wider range of biological models, geographical settings and temporal scales.

## Electronic supplementary material

Supplementary Information 1

Supplementary Information 2

Supplementary Information 3

Supplementary Information 4

Supplementary Information 5

Supplementary Information 6
