# Supplementary material for: Independent estimates of marine population connectivity are more concordant when accounting for uncertainties in larval origins
Source: Sci Rep. 2018 Feb 8;8:2641. doi: 10.1038/s41598-018-19833-w (PMC5805787; doi:10.1038/s41598-018-19833-w)
Supplement: Supplementary file 1 — Supplementary Information 1 [file 41598_2018_19833_MOESM1_ESM.pdf]

Supplementary Information 1: literature review

Independent estimates of marine population connectivity are more concordant when accounting for uncertainties in larval origins

Nolasco R[1,2], Gomes I[3,4], Peteiro L[3,5], Albuquerque R[3], Luna T[1], Dubert J[1], Swearer SE[6], Queiroga H[1]*

[1] Departamento de Física & CESAM - Centro de Estudos do Ambiente e do Mar, Universidade de Aveiro, 3810-193 Aveiro, Portugal
[2] Instituto de Investigacións Mariñas (CSIC), Eduardo Cabello 6, 36208 Vigo, Spain
[3] Departamento de Biologia & CESAM - Centro de Estudos do Ambiente e do Mar, Universidade de Aveiro, 3810-193 Aveiro, Portugal
[4] Marine Biology Research Group, Ghent University, 9000 Ghent, Belgium
[5] Coastal Ecology Research Group (EcoCost), Department of Ecology and Animal Biology, University of Vigo, Spain
[6] School of BioSciences, University of Melbourne, Parkville, Victoria, 3010, Australia

Corresponding author*
Henrique Queiroga: henrique.queiroga@ua.pt

We reviewed the literature on the use of multiple methodologies to estimate marine larval dispersal and connectivity matrices. In order to widen the search we used a double search strategy. First we used the following search key in Web of Science for the years 1990 to 2016: (ts=(larv* not insect*) or ts=(propagul* not plant)) and ts=(dispersal or connectivity) and (ts=((biophysical or oceanographic or hydrod*) and model) and ts=(genetic*) or ts=((biophysical or oceanographic or hydrod*) and model) and ts=(microchem* or geochem* or *elemental) or ts=(genetic*) and ts=(microchem* or geochem* or *elemental) or ts=(matrix or matrices)). This search retrieved 238 records. Then we added to this list all 332 papers citing Levin (2006), which was one of the first review and synthesis papers advocating the need to use a multiple methodological approach to the problem of larval dispersal estimates. Of the 507 unique matches identified, 41 papers conjugated at least two methodologies to estimate dispersal, with typical approaches employing a biophysical numerical model and either genetic markers or elemental fingerprinting, or genetic markers and elemental fingerprinting. In the analysis we retained papers that used this multiple approach independently of whether the data from the methodologies used were obtained from the same or from different studies. (Supplementary Table 1.1).

Definition of terms included in Supplementary Table 1.1

In the definitions below superscripts refer to entries in the first column of Supplementary Table 1.1.

Dispersal metrics: type of index used to evaluate genetic or demographic connectivity; usually in the form of a distance matrix (triangular) or of a connectivity matrix (square) among population units (but isolation-by-distance regression also considered); we considered that the matrices were effectively calculated even if the publication does not explicitly include them.

Uncertainty: whether the intrinsic uncertainty of the empirical method (genetics, elemental fingerprinting or current measurements) was accounted for in the assignment of individuals to the population units; intrinsic variability of the biophysical models is usually not assessed, or is assessed in previous independent studies not included in the reviewed papers, and was not included in the table; most studies that used

biophysical models did test different biological scenarios in order to bracket the uncertainty of the biological parameters, but this was also not included in the table.

Type of comparison: quantitative, when of some kind of test statistics of the fit between the dispersal estimated by the different approaches was calculated and assessed (e. g. correlation, Mantel test, likelihood ratio); semi-quantitative, when different approaches were tested separately, followed by a numerical comparison of the test statistics (e. g. magnitude of variability explained by separate isolation-by-distance lines); qualitative, when only a verbal description of the congruence of the different approaches is provided.

Dispersal probability matrix: quantifies the proportion of larvae released from each source population that survives to settle into any destination population (square matrix, normalized by the number of larvae that were released form each location; the diagonal of the matrix is the proportion of local retention).

Migration probability matrix: quantifies the proportion of settlers into each destination population that originates from a particular larval source (square matrix, normalized by the number of larvae that recruit into each location; the diagonal of the matrix is the proportion of self-recruitment).

Distance matrix: geographical, oceanographic or genetic distance between populations (triangular matrix).

Oceanographic distance: accounts for the transport induced by ocean currents (variably termed biophysical distance, derived oceanographic distance, oceanographic resistance, transport time or transport probability).

Genetics: all genetic analyses used microsatellites, except in [a] that used COI, [b] that used cytochrome b, [c] that used the control region, and [d] that used elongation factor 1 alfa; genetic distances calculated always among population units except in [f]; in [e], distances among cohorts also calculated; genetic distances calculated with a variety of indices (F_{ST}, G_{ST}, Jost's D_{est}, Nei's D_A); migration probability matrix calculated with parental assignment or with genetic assignment methods.

Model: biophysical numerical model coupling a baroclinic hydrodynamic, free-surface model (all 3D except [e], which is 2D) adjusted to local bathymetry (with variable degrees of scale, nesting, grid resolution and forcing), with a biological model describing spawning distribution and

larval biology (including different combinations of spawning periodicity and intensity, growth rate, mortality rate, competence period and larval behaviour).

Elemental chemistry: elemental composition of otoliths determined with LA-ICPMS; migration probability matrix calculated by obtaining an atlas of natal signatures and by assigning recruits to source populations using discriminant functions.

Current measurements: currents derived from maps of sea level anomaly assuming geostrophic equilibrium in [g], and measured with ADCP in [h].

Analysis

The two most common approaches have been to use genetic markers and a numerical biophysical model, or the microchemistry of hard parts and a numerical biophysical model, but genetic markers and micro-chemistry, and combinations of genetic markers or microchemistry with current measurements, have also been employed. Ideally, a migration probability matrix could be produced by each methodology, which would allow a spatially explicit estimate of intensity of dispersal and connectivity, and a direct numerical comparison of dispersal estimates. However, constraints associated with life-cycle traits, type of biological material and, presumably, available expertise and funding resulted in a variety of dispersal metrics employed by the different studies, which necessarily influenced the types of comparisons that could be made between estimates.

Genetic techniques most commonly employed estimated distance matrices among sampled populations based on a variety of indices (F_{ST}, G_{ST}, Jost's D_{est}, Nei's D_A) derived from microsatellites[16-18,22,23,26-31,34,35,38,40,42-44,48,49,51,53,54,61], COI[25,37], cytochrome b[21], the control region[33,36], or elongation factor 1 alfa[25], for a total of thirty three studies. Two studies calculated a multi-generation migration probability matrix using

coalescent analysis of gene flow based on microsatellites[22] or on COI[37]. Nine studies estimated a contemporary migration probability matrix using genetic[15,19,30,32,44,47,50] or parentage assignment tests[24,52], all based on microsatellites.

Studies using the microchemistry of hard parts (elemental fingerprinting) have been less commonly applied in conjunction with other approaches, likely because the technique can be applied to a much smaller range of organisms (typically fishes and bivalves). We identified only four studies that calculated a contemporary migration probability matrix[15,27,45,50], all applying discriminant function analysis to assign individuals to putative source populations. Four other studies used elemental fingerprinting to clarify similarities among cohorts[33], classify self-recruits *versus* dispersers[24] and freshwater dispersers *versus* marine dispersers[19], or to characterize source locations[36].

Biophysical numerical modelling was the technique most commonly used in tandem with a second (or third) approach to investigate connectivity (35 out of the 40 studies). Biophysical models coupled a baroclinic hydrodynamic model adjusted to local bathymetry, with a biological model describing spawning distribution and larval biology. The oceanographic models differed in spatial scale, nesting, grid resolution and forcing. In all cases different combinations of spawning periodicity and intensity, growth rate, mortality rate, competency period and/or larval behaviour were employed, in an attempt to bracket the uncertainty regarding parameterization of these processes. Simulated periods were chosen to cover the expected sources of temporal variability, whereas some kind of temporal integration was typically applied in order to calculate an average connectivity matrix. Twenty one studies estimated dispersal probability matrices[16-18,20-23,25,30,32,34,35,37,38,41,42,44,46,47,51], while fourteen studies calculated migration probability matrices[26,28,29,31,39,40,43,48-50,52-55].

Advancements on the merging of independent approaches to describe dispersal patterns have been to use connectivity matrices predicted by biophysical models (either dispersal probability matrices or migration probability matrices) into population genetic models, in order to predict genetic structure. If the predicted genetic structure matches the observed structure, a case is made that migration mediated by oceanographic

patterns of propagule transport influences gene flow. Five studies used a derivation of the Bodmer & Cavalli-Sforza matrix model of migration[58] to predict equilibrium allele frequencies after a variable number of generations, and compared these with observations through correlation between distance matrices[40,54], comparison between isolation-by-distance and isolation-by-oceanographic distance statistics tested separately[29], or qualitatively[26,31]. Similar approaches, where modelled pairwise migration probabilities were used to inform a population model predicting allele frequencies at equilibrium, were applied by another three studies, which used Mantel tests to compare matrices of observed and predicted genetic distances[48], sums of squared differences between predicted and observed allele frequencies[25], or qualitative assessments of fit[17] to compare predicted and observed genetic structure.

Supplementary Table 1.1. Methodologies employed by studies that used a multiple approach to the estimation of marine larval dispersal and connectivity matrices.

Approaches	Study taxa	Location	Dispersal metrics	Uncertainty	Type of comparison	Reference
Genetics and Model	Giant kelp, *Macrocystis pyrifera*	Santa Barbara Channel, California	Genetic distance matrix; migration probability matrix calculated using genetic assignment; modeled dispersal probability matrix used to estimate probability-weighted mean transport time	Not accounted	Semi-quantitative; comparison of the percentage variability explained by trends of isolation-by-distance and isolation-by-oceanographic distance	[30]
Genetics and Model	Coral, *Acropora palmata*	Caribbean	Genetic distance matrix; modeled dispersal probability	Not accounted	Qualitative	[16]

Approaches	Study taxa	Location	Dispersal metrics	Uncertainty	Type of comparison	Reference
			matrix			
Genetics and Model	Coral reef fish, *Glaucosoma hebraicum*	Western Australia	Genetic distance matrix; modeled dispersal probability matrix; modeled transport probability	Not accounted	Quantitative; Mantel test used to compare matrices of observed and predicted distances	[34]
Genetics and Model	Coral reef fish, *Lethrinus nebulosus*	Northwestern Australia	Genetic distance matrix; modeled dispersal probability matrix	Not accounted	Qualitative	[35]
Genetics and Model	Common cockle, *Cerastoderma edule*	Southern Irish Sea	Genetic distance matrix; modeled dispersal probability matrix;	Not accounted	Qualitative	[42]
Genetics [a] and Model	Marine gastropods, *Nerita plicata* and *Nerita albicilla;* amphidromous gastropods *Neritina canalis* and *Neritina dilatatum*	Indo-Pacific	Coalescent analysis of gene flow fed with modeled dispersal probability matrix to predict observed genetic structure	Not accounted	Quantitative; log Bayes factors analysis that the predicted genetic structure fits the observed genetic structure	[37]
Genetics and	Corals, *Acropora*	Greater	Genetic distance matrix;	Not accounted	Semi-quantitative; comparison	[49]

Approaches	Study taxa	Location	Dispersal metrics	Uncertainty	Type of comparison	Reference
Model	*hyacinthus* and *A. digitifera*	Micronesia	modeled migration probability matrix transformed into oceanographic distance		of the percentage variability explained by trends of isolation-by-distance and isolation-by-oceanographic distance	
Genetics and Model	White sea bream, *Diplodus sargus sargus*	Apulian coast, Adriatic	Genetic distance matrix; modeled dispersal probability matrix	Not accounted	Qualitative	[38]
Genetics and Model	Shore crab, *Carcinus maenas*	Western Iberian Peninsula	Genetic distance matrix; modeled migration probability matrix	Not accounted	Qualitative	[39]
Genetics [e] and Model	Gastropod, *Crepidula fornicata*	English Channel	Genetic distance matrix; modeled dispersal probability matrix; modeled transport probability matrix	Not accounted	Quantitative; Mantel test used to compare observed and predicted distance matrices	[18]
Genetics and Model	Coral, *Montastraea annularis*	Caribbean	Genetic distance matrix; modeled migration probability matrix used to project allele frequencies after 100 generations based on Bodmer & Cavalli-Sforza (1968) matrix model of migration	Not accounted	Quantitative; non-parametric correlation between distance matrices	[40]

Approaches	Study taxa	Location	Dispersal metrics	Uncertainty	Type of comparison	Reference
Genetics and Model	Coral, *Acropora cervicornis*	Caribbean	Genetic distance matrix from previous study; modeled dispersal probability matrix fed into a population genetic model to simulate multi-generations genetic distance based on 10 independent loci	Not accounted	Qualitative	[17]
Genetics [a, d] and Model	Barnacle, *Balanus glandula*	California	Observed allele frequencies; modeled migration probability matrix used to estimate deterministic transitions of allele frequencies after 200 generations	Not accounted	Quantitative; sum of squared differences between predicted and observed allele frequencies	[25]
Genetics and Model	Horse mussel, *Modiolus modiolus*	Irish Sea	Genetic distance matrix; modeled dispersal probability matrix	Not accounted	Qualitative	[51]
Genetics and Model	Polychaete, *Pectinaria koreni*	English Channel, British Islands, North Sea	Coalescent analysis of gene flow; genetic distance matrix; modeled multi-generation dispersal probability matrix	Not accounted	Qualitative	[22]
Genetics [e]	Polychaete,	Seine Bay	Genetic distance matrix;	Not accounted	Qualitative	[43]

Approaches	Study taxa	Location	Dispersal metrics	Uncertainty	Type of comparison	Reference
and Model	*Pectinaria koreni*		modeled migration probability matrix			
Genetics and Model	Model coral species	Caribbean	Genetic distance matrices from previous studies; modeled migration probability matrix used to project allele frequencies after 100 generations based on Bodmer & Cavalli-Sforza (1968) matrix model of migration	Not accounted	Qualitative	[26]
Genetics and Model	Model coral species	Indo-West Pacific	Genetic distance matrices from previous studies; modeled migration probability matrix used to project allele frequencies after 100 generations based on Bodmer & Cavalli-Sforza (1968) matrix model of migration	Not accounted	Qualitative	[31]
Genetics and Model	Shore crab, *Carcinus maenas*	North Sea, Kattegat, Skagerrak	Genetic distance matrices from previous studies; modeled dispersal single- generation and multiple-generation probability	Not accounted	Qualitative	[55]

Approaches	Study taxa	Location	Dispersal metrics	Uncertainty	Type of comparison	Reference
			matrix			
Genetics and Model	Anemonefish, *Amphiprion bicinctus*	Central Red Sea	Migration probability matrix calculated using parentage assignment; modeled self-retention and self-recruitment	Not accounted	Qualitative	[52]
Genetics and Model	Abalone, *Haliotis coccoradiata*	New South Wales, Australia	Genetic distance matrix; modeled dispersal probability matrix	Not accounted	Qualitative	[20]
Genetics and Model	Bicolor damselfish, *Stegastes partitus*	Central American Gulf coast	Genetic distance matrix; modeled dispersal probability matrix	Not accounted	Qualitative	[23]
Genetics and Model	Mediterranean shore crab, *Carcinus aestuarii*	Adriatic	Genetic distance matrix and Bayesian clustering; modeled dispersal probability matrix	Not accounted	Qualitative	[46]
Genetics and Model	Fish, *Serranus cabrilla*	Mediterranean	Migration probability matrix calculated using genetic assignment; modeled dispersal probability matrix	Accounted using a posterior probability threshold of 0.80 for correct assignment to parental population	Quantitative; correlation between connectivity matrices	[32]
Genetics and	Clownfish,	Oman,	Migration probability matrix	Accounted using a	Qualitative	[47]

Approaches	Study taxa	Location	Dispersal metrics	Uncertainty	Type of comparison	Reference
Model	*Amphiprion omanensis*	Arabic Sea	calculated using genetic assignment; modeled dispersal probability matrix	posterior probability threshold of 0.95 for correct assignment to parental population		
Genetics and Model	Rock scallop, *Spondylus calcifer*	Northern Gulf of California	Genetic distance matrix; modeled dispersal probability matrix	Not accounted	Qualitative	[41]
Genetics and Model	Bat star, *Patiria miniata*	Nothwester n Pacific	Genetic distance matrices from a previous study; modeled dispersal probability matrix fed into a genetic population model to simulate multi-generations genetic distance based on 50 independent loci	Not accounted	Quantitative; Mantel test used to compare matrices of observed and predicted genetic distances	[48]
Genetics and Model	Rock lobster, *Jasus edwardsii*	Tasmanian Sea	Migration probability matrix calculated using genetic assignment; modeled dispersal probability matrix	Not accounted	Qualitative	[44]
Genetics and Model	Coral, *Acropora spicifera*	Houtman Abrolhos Islands, Western	Genetic distance matrix; modeled migration probability matrix projected forward for 10 generations used to calculate	Not accounted	Semi-quantitative; significance of isolation-by-distance and isolation-by-oceanographic distance tested separately	[53]

Approaches	Study taxa	Location	Dispersal metrics	Uncertainty	Type of comparison	Reference
		Australia	oceanographic distance			
Genetics and Model	Whelk, *Kelletia kelleti*	California	Genetic distance matrix; modeled migration probability matrix used to project allele frequencies after 1000 generations based on Bodmer & Cavalli-Sforza (1968) matrix model of migration and used to estimate oceanographic distance	Not accounted	Semi-quantitative; significance of isolation-by-distance and isolation-by-oceanographic distance tested separately	[29]
Genetics and Model	Fish *Champsocephalus gunnari*, fish *Notothenia rossii*	Scotia Sea, Antarctic	Genetic distance matrix; modeled migration probability matrix used to project allele frequencies after a number of generations producing a level of genetic differentiation identical to the observed level, based on a modified Bodmer & Cavalli-Sforza (1968) matrix model of migration	Not accounted	Quantitative; correlation of the observed genetic distance with predicted genetic distance	[54]
Genetics, Model and	Kelp bass *Paralabrax*	Southern California	Genetic distance matrix; modeled migration probability	Not accounted	Quantitative; used linear multiple regression to predict	[28]

Approaches	Study taxa	Location	Dispersal metrics	Uncertainty	Type of comparison	Reference
Environmental variables	*clathratus*, Kellet's whelk *Kelletia kelletii*, California spiny lobster *Panulirus interruptus*	Bight	matrix used as a metric of oceanographic distance		the effects of oceanographic distance and other environmental variables on genetic distance	
Genetics [c, e, f] and Elemental chemistry	Coral reef fish, *Neopomacentrus mirya* and *Chromis viridis*	Red Sea	None; study wasn't directly about dispersal but about genetic and/or chemical similarity among settling cohorts; measures were genetic and elemental similarity matrices	Not accounted	Qualitative	[33]
Genetics and Elemental chemistry	Coral reef fish, *Amphiprion percula*	Papua New Guinea	None; individuals classified as self-recruiters versus dispersers based on parentage assignement	Not accounted	Qualitative; repeated measures MANOVA with individuals assigned to groups based on parentage	[24]
Genetics and Elemental chemistry	Rainbow smelt, *Osmerus mordax*	Newfoundland, Canada	Migration probability matrix calculated using genetic assignment; elemental signatures classified as freshwater, estuarine or marine	Not accounted	Qualitative	[19]

Approaches	Study taxa	Location	Dispersal metrics	Uncertainty	Type of comparison	Reference
			to estimate levels of self-recruitment versus straying among tributaries			
Genetics [c] and Elemental chemistry	European conger eel *Conger conger*	North-east Atlantic and Western Mediterranean	Genetic distance matrix; elemental chemistry only used for discrimination of core signatures among locations	Not accounted	Qualitative	[36]
Genetics, Elemental chemistry and Model	Yellow perch, *Perca flavescens*	Lake Erie, USA	Migration probability matrix calculated using genetic assignment; migration probability matrix calculated using and elemental assignment; hydrodynamic model used to reduce uncertainty in the origins of larvae used to characterize source population signatures	Accounted using a posterior probability threshold of 0.70 for correct assignment to source population for both genetic and elemental data	Qualitative	[50]
Genetics and Elemental chemistry	Neon damsel, *Pomacentrus coelestis*	Northern West Pacific	Genetic distance matrix; migration probability matrix calculated using elemental assignment	Not accounted	Qualitative	[27]

Approaches	Study taxa	Location	Dispersal metrics	Uncertainty	Type of comparison	Reference
Genetics and Elemental chemistry	Black rockfish, *Sebastes melanops*	Oregon and Washington, USA	Genetic distance matrix; migration probability matrix calculated using genetic assignment; migration probability matrix calculated using elemental assignment	Not accounted	Qualitative	[15]
Genetics [b] and Current measurements	Limpet, *Cellana strigilis*	New Zealand, Chatham and Sub-Antarctic Islands	Genetic distance matrix; modeled dispersal probability matrix used to estimate transport times	Not accounted	Qualitative	[21]
Elemental chemistry and Current measurements	Reef fish, *Hypsypops rubicundus*	California	Migration probability matrix calculated using elemental assignment; estimated dispersal distances	Not accounted	Qualitative	[45]

References

The references are listed in the main text.
