# Supplementary material for: Independent estimates of marine population connectivity are more concordant when accounting for uncertainties in larval origins
Source: Sci Rep. 2018 Feb 8;8:2641. doi: 10.1038/s41598-018-19833-w (PMC5805787; doi:10.1038/s41598-018-19833-w)
Supplement: Supplementary file 2 — Supplementary Information 2 [file 41598_2018_19833_MOESM2_ESM.pdf]

Supplementary information 2: biophysical numerical model

Independent estimates of marine population connectivity are more concordant when accounting for uncertainties in larval origins

Nolasco R[1,2], Gomes I[3,4], Peteiro L[3,5], Albuquerque R[3], Luna T[1], Dubert J[1], Swearer SE[6], Queiroga H[1]*

[1] Departamento de Física & CESAM - Centro de Estudos do Ambiente e do Mar, Universidade de Aveiro, 3810-193 Aveiro, Portugal
[2] Instituto de Investigacións Mariñas (CSIC), Eduardo Cabello 6, 36208 Vigo, Spain
[3] Departamento de Biologia & CESAM - Centro de Estudos do Ambiente e do Mar, Universidade de Aveiro, 3810-193 Aveiro, Portugal
[4] Marine Biology Research Group, Ghent University, 9000 Ghent, Belgium
[5] Coastal Ecology Research Group (EcoCost), Department of Ecology and Animal Biology, University of Vigo, Spain
[6] School of BioSciences, University of Melbourne, Parkville, Victoria, 3010, Australia

Corresponding author*
Henrique Queiroga: henrique.queiroga@ua.pt

The target species

Mytilus galloprovincialis has a complex life cycle with a planktonic larval stage and sessile juvenile and adult phases. *M. galloprovincialis* can release and fertilize gametes during the whole year at intermediate latitudes, but massive spawnings are concentrated between early spring and summer, with an additional spawning peak of smaller magnitude in autumn[66,70,71]. The planktonic larval stage has an estimated duration ranging from 2 to 6 weeks depending mostly

on temperature and food concentration[67,68,72-74]. Due to limited larval swimming capacity (≤ 0.1 cm s^{-1} for bivalves[75]), it has been traditionally assumed that larval dispersal patterns are mostly dependent on pelagic larval duration (PLD), survival and hydrographic patterns. However, vertical migrations between layers flowing in opposite directions have been reported as a mechanism that enables larvae to regulate along- and cross-shore displacements[76]. Larval behaviour is a key component of larval dispersal but not completely understood[77]. According to some authors mussel larvae concentrate at the surface during the first developmental stage (veliger) but tend to loose upward swimming velocity as development progress (pediveliger[78]). Light and discontinuities in the water column, like pycnoclines or food patches, are commonly suggested as relevant factors altering vertical migration behaviour[79-81] and, in general, larvae capacity for vertical position regulation might be limited to slow current speeds and low turbulence[78,81].

Several aspects of the species' reproductive biology were explicitly simulated by the Lagrangian component (see below) of the model, including spatial and temporal distribution of spawning intensity, larval vertical migration, larval growth rate and mortality. Four spawning scenarios (see main text) were simulated, all accounting for differences in mussel density along rocky shore habitats of the western Iberian coast[62] but differing on spawning periodicity in order to bracket the seasonal variability[66]. Two larval behaviours were also modelled according to available information (see main text): passive behaviour[82] and ontogenetic vertical migration[78,83]. An unrealistic third behaviour, where larvae were forced to dwell in the bottom layer, was included in order to provide a contrast to the other two scenarios. Temperature-dependent planktonic larval duration (PLD) and mortality were modelled pooling information from laboratory studies[84,85].

The oceanographic model

The simulations were conducted using a 3-D free-surface, terrain-following primitive equation hydrostatic model configurable for fully realistic regional applications, based on the Regional Ocean Modelling System[86]. The present configuration represents an improvement and extension of the configuration used by[87,88] to the Atlantic margin of the Iberian Peninsula, and was applied by[39] to describe the links between dispersal and supply of *Carcinus maenas* larvae to the Ria de

Aveiro, and by[89] to the study of the *Carcinus maenas* larval connectivity along the Western Iberian Margin (WIM hereinafter).

Three grids were used to resolve the circulation of the WIM (Supplementary Fig. 2.1): a large domain (LD), a medium domain (MD) and a small domain (SD). The LD, from 12.5° W to 5.5° W and 34.4° N to 45.5° N, has a grid resolution of 1/27° (*ca.* 3 km) and 60 vertical levels. This domain has been used to study the ocean circulation in the WIM by[90], and provides initial and boundary conditions, through offline nesting, to the MD domain. The MD has a horizontal resolution of 1/60° (*ca.* 1,4 km) and 45 vertical levels, extending from the Cape St Vincent at 37° N to Cape Finisterre, 43° N, and from 11.5° W to the WIM coast at 8.5° W. The MD covers an area of ~670 x 245 km and constitutes the target domain used for the dispersal simulations. The SD, with a grid resolution of 1/180° (*ca.* 450 m) and 45 levels, was implemented in order to solve the details of the circulation in the main region where natal and recruit signatures were collected, including the Cascais and Arrábida bays. The SD domain exchange information with MD through two-way nesting, improving the circulation in the target domain MD. Tidal elevation and current ellipses, from TPXO global tide model, was applied at the boundaries of the MD (and hence SD) in order to solve the tidal dynamics in both domains. The model was run from the 1st of January until the 30th of July, 2013, with an atmospheric forcing resulting from the outputs from the Weather Research and Forecasting model (WRF, [92]), which was run for the same period with a 3 km resolution, and applied to LD, MD and SD domains. The inflow of freshwater to the ocean, originated from the main rivers of the region, was included in the form of realistic river outflow (provided by INAG, Water Institute of Portugal), for all the three domains. The outputs of the model, consisting of temperature, salinity, and three-dimensional velocity fields, were stored every hour in order to be used for the Lagrangian model described below.

The performance of the LD concerning the hydrology and current fields was evaluated elsewhere[90]. In the present study a validation of the oceanographic model was obtained by visually comparing (Supplementary Fig. 2.2) sea surface temperature (SST) fields predicted for the MD, during the period covered by the study, with satellite data retrieved from the Advanced Very High Resolution Radiometer (made available by the EUMETSAT Ocean & Sea Ice Satellite Application Facility). The main features of the circulation are captured by the model, including upwelled water and filaments during the first part of the study and the poleward flow of a

warmer water mass during an extended period of upwelling relaxation that started at the beginning of July.

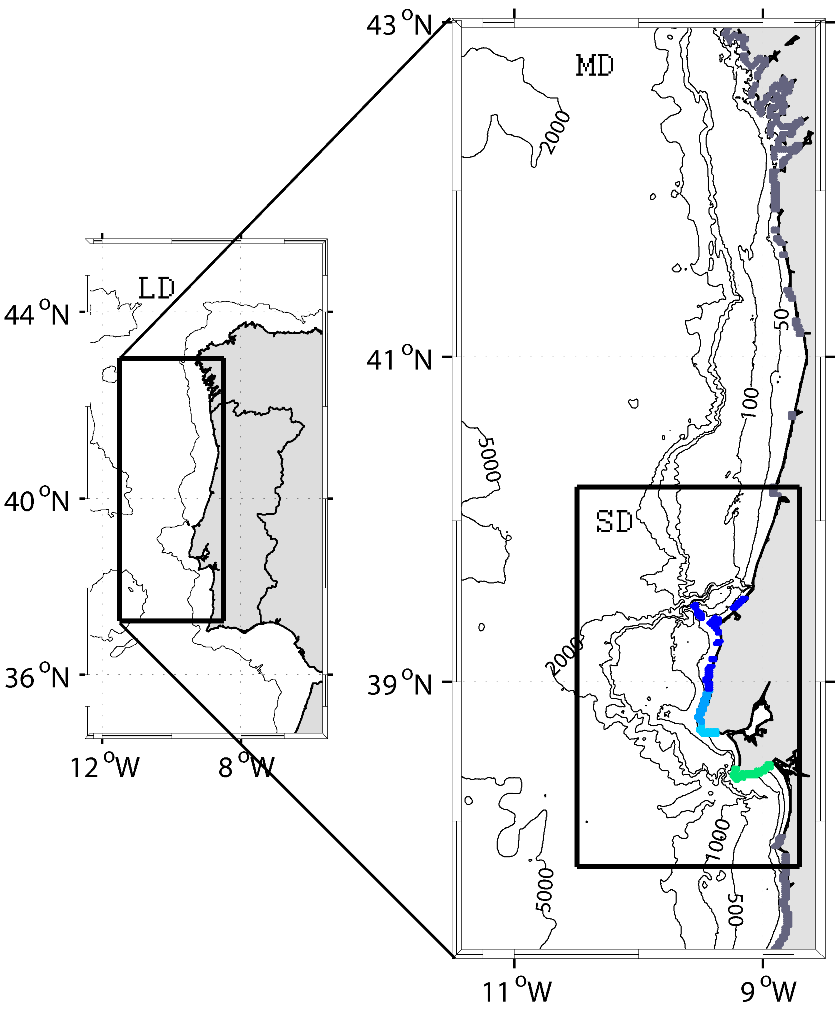

Supplementary Figure 2.1. Map of the region showing the large (LD), medium (MD) and small (SD) domains. At the right panel, the MD shows the 50, 100, 500, 1000, 2000 and 5000 m bathymetric contours. The locations of rocky shore where emission/recruitment were simulated are represented in green/blue for the sampling region, and in grey for the remaining domain. This figure was produced using Python V 2.7.2 (www.python.org).

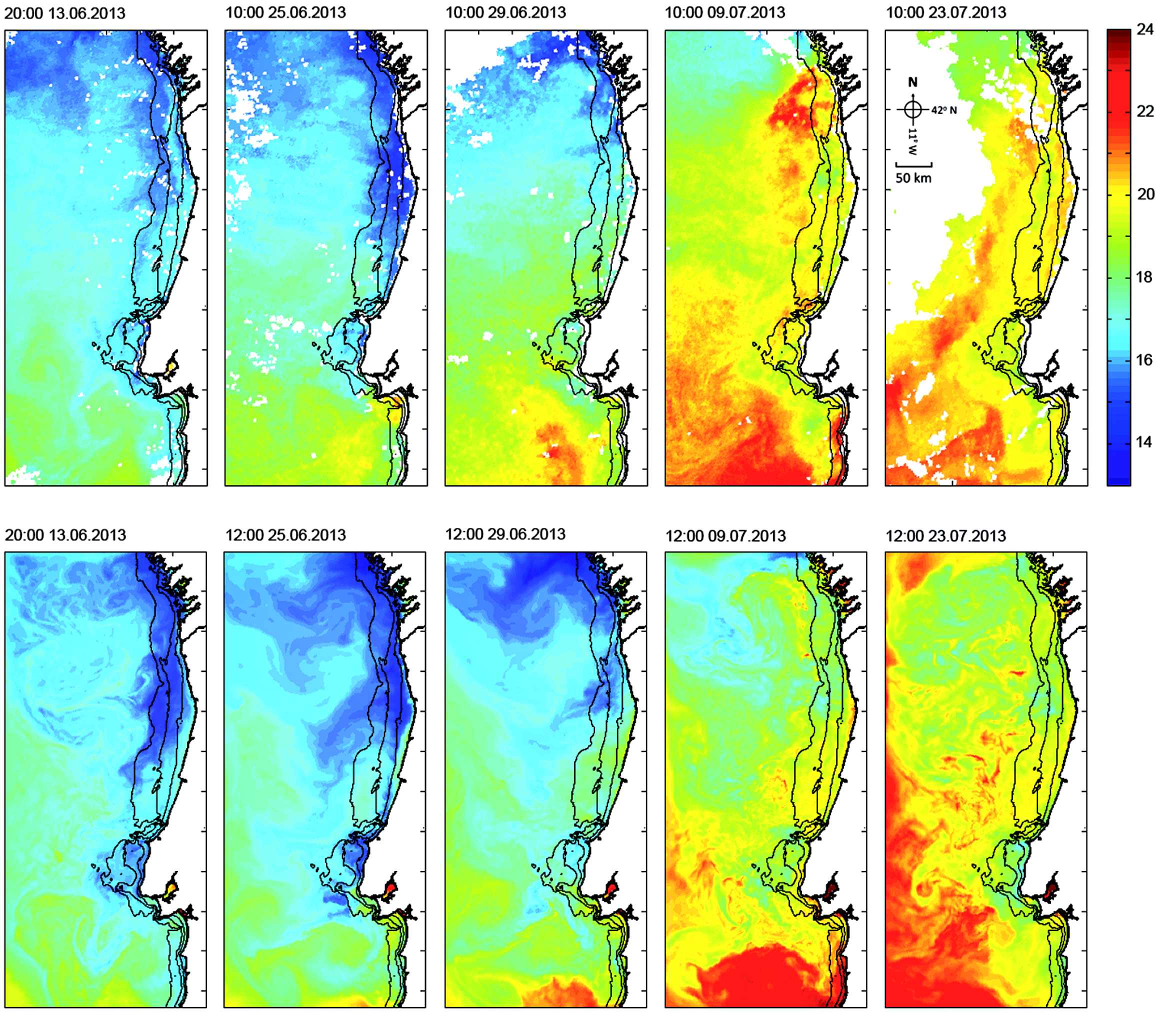

Supplementary Figure 2.2. Satellite (top row) and MD (bottom row) SST for days 13, 25 and 29 of June, and 9 and 23 of July, 2013. The colour bar (SST, ° C) is the same for all the images. This figure was produced using Python V 2.7.2 (www.python.org).

The biological and Lagrangian offline model

In order to simulate spawning, behaviour and growth of *M. galloprovincialis* larvae an Individual Based Model (IBM) was coupled to ROMS using ROFF[93]. ROFF is a drifter-tracking code that simulates larval trajectories from stored ROMS velocity and hydrological fields. The drifter-tracking code simulates larval trajectories from stored ROMS velocity and hydrological fields using a high order predictor corrector scheme to integrate the motion equation

$dX/dt = U_{roms}(X,t)$, with X being the position vector (x,y,z), and U_{roms} being the modelled 3D velocity vector over time, given an initial condition $X(t_0) = X_0$. The time step used in this Lagrangian model, dt, is 300s. Additionally to the advection generated by the model velocities, the particle movements included random velocities in the vertical direction, which were used to parameterize unresolved turbulent processes.

Spawning and settlement were simulated along rocky coasts (Supplementary Fig. 2.1) using a coastal buffer strip of 2 cells for spawning and 3 cells for settlement. Spawning was made proportional to the mussel biomass at each segment of the coast[65] and to seasonal spawning activity[66]. This was accomplished by spawning into the model, during predicted high water, a number of virtual larvae proportional to biomass, and by varying the frequency of tides when spawning was simulated (from every high water at full spawning intensity, through 1 in every 3rd high water at low spawning intensity, to no spawning; see main text for spawning scenarios). Passive larvae were advected according to the 3D current velocities predicted by the oceanographic model. Vertically migrating larvae were forced to change layer instantaneously once the appropriate age was reached, in the case of ontogenetic behaviour, and immediately after spawning, in the case of bottom dwelling larvae, and advected at the current velocities at the respective level.

The proportional effects of temperature on PLD, and of temperature and salinity on mortality, based on the time a larva was exposed to a specific temperature in the case of PLD, or to a specific combination of temperature and salinity in the case of mortality, were estimated by linearly interpolating between the laboratory data for each larval stage. Age and the probability of death were assessed at each time step of the Lagrangian model. Larvae were killed randomly based on the proportional death rate during that time step. If a larva survived physiological stress it would grow from age 0 at spawning to age 1 at veliger and age 2 at pediveliger stages; pediveligers lived and remained competent until age 3 and then died. No other temporally or spatially distributed source of mortality (e.g. predation) was used because of lack of information.

Twelve runs of the model were performed, corresponding to four spawning scenarios and three larval behaviours. In each run, 184.340 larvae were simulated.

References

The references are listed in the main text.
