# Supplementary material for: Independent estimates of marine population connectivity are more concordant when accounting for uncertainties in larval origins
Source: Sci Rep. 2018 Feb 8;8:2641. doi: 10.1038/s41598-018-19833-w (PMC5805787; doi:10.1038/s41598-018-19833-w)
Supplement: Supplementary file 3 — Supplementary Information 3 [file 41598_2018_19833_MOESM3_ESM.pdf]

Supplementary Information 3: matrix correlations

Independent estimates of marine population connectivity are more concordant when accounting for uncertainties in larval origins

Nolasco R[1,2], Gomes I[3,4], Peteiro L[3,5], Albuquerque R[3], Luna T[1], Dubert J[1], Swearer SE[6], Queiroga H[1]*

[1] Departamento de Física & CESAM - Centro de Estudos do Ambiente e do Mar, Universidade de Aveiro, 3810-193 Aveiro, Portugal
[2] Instituto de Investigaciónes Mariñas (CSIC), Eduardo Cabello 6, 36208 Vigo, Spain
[3] Departamento de Biologia & CESAM - Centro de Estudos do Ambiente e do Mar, Universidade de Aveiro, 3810-193 Aveiro, Portugal
[4] Marine Biology Research Group, Ghent University, 9000 Ghent, Belgium
[5] Coastal Ecology Research Group (EcoCost), Department of Ecology and Animal Biology, University of Vigo, Spain
[6] School of BioSciences, University of Melbourne, Parkville, Victoria, 3010, Australia

Corresponding author*
Henrique Queiroga: henrique.queiroga@ua.pt

Supplementary Table 3.1. Pearson correlation coefficients between observed and modelled connectivity matrices for different combinations of larval behaviour, spawning regime, partitioning of the core region, and confidence level of the assignment of recruits into source populations. Sections A and B refer to the core matrices (without unknown row), sections C, D and E refer to the core matrices plus unknown row. A: uncorrected modelled matrix. B and C: modelled matrix corrected for Type 2 recruits. D: modelled matrix corrected for Type 3 recruits. E: modelled matrix corrected for Type 2 and Type 3 recruits. Shading indicates strength of the correlation; bold values indicate the highest correlation in each section. Pa= passive larvae. Om= larvae migrating ontogenetically. Bl= larvae dwelling in the bottom layer. S1= continuous larval emission during each high tide until July 12. S2= continuous larval emission during each high tide until June 30, then larval emission skipping one of every two high tides until July 12. S3= continuous larval emission during each high tide until June 30, then larval emission skipping two of every three high tides until July 12. S4= Continuous larval emission during each high tide until July 1, no more larvae released afterwards. 3x3, 3x4 and 4x4= spatial organization of the core region into 3 or 4 origin x destination cells. Better= recruits assigned into an origin when the probability of pertaining to that origin is better than that of pertaining to any other origin. %99, %95, %90, %75, %50= recruits assigned into an origin when the probability of pertaining to that origin is larger than the level indicated.

A

CORE MATRICES (without unkown row); uncorrected modelled matrix		Larval behaviour / Spawning regime combinations											
		PaS1	PaS2	PaS3	PaS4	OmS1	OmS2	OmS3	OmS4	BlS1	BlS2	BlS3	BlS4
Partitioning of the core region / Confidence level combinations	3x3%99	0.64	0.72	0.77	0.87	0.58	0.67	0.75	0.82	0.28	0.31	0.34	0.38
	3x3%95	0.77	0.84	0.88	0.94	0.71	0.79	0.86	0.90	0.41	0.44	0.47	0.51
	3x3%90	0.77	0.84	0.88	0.94	0.72	0.80	0.86	0.91	0.41	0.44	0.47	0.50
	3x3%75	0.80	0.86	0.90	**0.96**	0.74	0.82	0.88	0.92	0.45	0.49	0.52	0.55
	3x3%50	0.79	0.85	0.89	0.94	0.72	0.80	0.87	0.91	0.41	0.44	0.47	0.51
	3x3 Better	0.77	0.83	0.88	0.93	0.70	0.78	0.85	0.89	0.38	0.42	0.45	0.49
	3x4%99	0.41	0.48	0.53	0.63	0.40	0.49	0.56	0.61	0.15	0.17	0.19	0.22
	3x4%95	0.61	0.67	0.71	0.79	0.59	0.67	0.73	0.78	0.33	0.36	0.38	0.41
	3x4%90	0.62	0.69	0.72	0.80	0.60	0.68	0.75	0.79	0.34	0.37	0.39	0.41
	3x4%75	0.62	0.68	0.72	0.79	0.61	0.69	0.74	0.77	0.37	0.39	0.42	0.44
	3x4%50	0.59	0.65	0.69	0.75	0.58	0.66	0.72	0.74	0.32	0.35	0.37	0.40
	3x4 Better	0.56	0.62	0.66	0.72	0.55	0.63	0.69	0.71	0.29	0.31	0.34	0.36
	4x4%99	0.40	0.47	0.50	0.60	0.38	0.46	0.52	0.56	0.13	0.15	0.17	0.19
	4x4%95	0.42	0.49	0.53	0.61	0.41	0.49	0.55	0.58	0.16	0.18	0.20	0.22
	4x4%90	0.52	0.58	0.61	0.68	0.51	0.57	0.62	0.63	0.25	0.26	0.29	0.29
	4x4%75	0.63	0.68	0.71	0.76	0.61	0.66	0.70	0.68	0.35	0.36	0.38	0.37
	4x4%50	0.66	0.71	0.73	0.78	0.64	0.69	0.73	0.70	0.38	0.38	0.41	0.40
	4x4 Better	0.65	0.70	0.72	0.76	0.64	0.69	0.72	0.69	0.37	0.38	0.40	0.38

B

CORE MATRICES (without unkown row); modelled matrix corrected for Type 2 recruits		Larval behaviour / Spawning regime combinations											
		PaS1	PaS2	PaS3	PaS4	OmS1	OmS2	OmS3	OmS4	BlS1	BlS2	BlS3	BlS4
Partitioning of the core region / Confidence level combinations	3x3%99	0.61	0.69	0.74	0.84	0.55	0.64	0.71	0.78	0.31	0.34	0.37	0.41
	3x3%95	0.75	0.81	0.86	0.93	0.69	0.77	0.82	0.88	0.44	0.47	0.50	0.54
	3x3%90	0.75	0.82	0.86	0.93	0.69	0.78	0.83	0.88	0.44	0.47	0.50	0.54
	3x3%75	0.78	0.84	0.88	**0.94**	0.71	0.80	0.84	0.89	0.49	0.52	0.55	0.59
	3x3%50	0.77	0.83	0.87	0.93	0.70	0.78	0.84	0.88	0.45	0.48	0.51	0.55
	3x3 Better	0.75	0.81	0.86	0.92	0.68	0.76	0.82	0.87	0.42	0.46	0.49	0.53
	3x4%99	0.39	0.46	0.50	0.61	0.37	0.46	0.51	0.57	0.17	0.19	0.21	0.24
	3x4%95	0.59	0.65	0.69	0.78	0.56	0.64	0.70	0.75	0.36	0.38	0.40	0.43
	3x4%90	0.61	0.67	0.70	0.79	0.58	0.66	0.71	0.76	0.37	0.39	0.41	0.44
	3x4%75	0.61	0.67	0.70	0.78	0.58	0.66	0.71	0.75	0.39	0.42	0.44	0.47
	3x4%50	0.58	0.64	0.68	0.75	0.56	0.64	0.69	0.72	0.35	0.38	0.40	0.43
	3x4 Better	0.55	0.61	0.65	0.72	0.53	0.61	0.65	0.69	0.32	0.34	0.37	0.39
	4x4%99	0.48	0.54	0.57	0.65	0.48	0.55	0.61	0.62	0.17	0.19	0.21	0.23
	4x4%95	0.50	0.56	0.59	0.66	0.51	0.58	0.63	0.64	0.19	0.22	0.24	0.26
	4x4%90	0.57	0.63	0.64	0.70	0.58	0.63	0.68	0.67	0.26	0.28	0.31	0.31
	4x4%75	0.64	0.69	0.70	0.74	0.65	0.69	0.73	0.70	0.34	0.36	0.38	0.38
	4x4%50	0.66	0.71	0.71	0.75	0.67	0.71	0.74	0.70	0.37	0.38	0.41	0.40
	4x4 Better	0.65	0.69	0.70	0.73	0.66	0.70	0.73	0.69	0.35	0.37	0.39	0.39

C

CORE MATRICES + UNKNOWNS; modelled matrix corrected for Type 2 recruits		Larval behaviour / Spawning regime combinations											
		PaS1	PaS2	PaS3	PaS4	OmS1	OmS2	OmS3	OmS4	BlS1	BlS2	BlS3	BlS4
Partitioning of the core region / Confidence level combinations	3x3%99	0.29	0.36	0.41	0.50	0.28	0.35	0.39	0.45	0.06	0.08	0.10	0.12
	3x3%95	0.48	0.56	0.61	0.70	0.47	0.54	0.59	0.64	0.19	0.22	0.24	0.27
	3x3%90	0.61	0.68	0.74	0.82	0.59	0.67	0.72	0.77	0.26	0.29	0.31	0.34
	3x3%75	0.71	0.78	0.83	**0.90**	0.69	0.77	0.81	0.85	0.37	0.39	0.42	0.45
	3x3%50	0.68	0.74	0.78	0.83	0.66	0.72	0.77	0.79	0.35	0.38	0.40	0.43
	3x3 Better	0.66	0.71	0.75	0.80	0.64	0.70	0.74	0.76	0.34	0.36	0.39	0.41
	3x4%99	0.13	0.19	0.23	0.32	0.14	0.19	0.24	0.29	-0.06	-0.06	-0.04	-0.03
	3x4%95	0.32	0.39	0.44	0.54	0.31	0.38	0.44	0.49	0.07	0.09	0.10	0.12
	3x4%90	0.44	0.52	0.57	0.68	0.42	0.50	0.58	0.64	0.16	0.18	0.19	0.21
	3x4%75	0.58	0.65	0.70	0.80	0.55	0.63	0.70	0.76	0.28	0.30	0.32	0.33
	3x4%50	0.57	0.64	0.67	0.74	0.54	0.61	0.67	0.71	0.30	0.32	0.34	0.35
	3x4 Better	0.55	0.60	0.64	0.71	0.52	0.58	0.64	0.68	0.28	0.30	0.32	0.33
	4x4%99	0.59	0.60	0.60	0.61	0.55	0.56	0.57	0.56	0.46	0.45	0.47	0.42
	4x4%95	0.64	0.67	0.67	0.70	0.60	0.63	0.65	0.66	0.47	0.46	0.48	0.44
	4x4%90	0.68	0.72	0.73	0.77	0.65	0.68	0.72	0.73	0.46	0.46	0.48	0.44
	4x4%75	0.72	0.77	0.79	0.84	0.69	0.73	0.78	0.80	0.44	0.44	0.47	0.43
	4x4%50	0.54	0.60	0.64	0.72	0.52	0.58	0.63	0.68	0.22	0.24	0.26	0.25
	4x4 Better	0.66	0.71	0.75	0.80	0.64	0.70	0.74	0.76	0.34	0.36	0.39	0.41

D

CORE MATRICES + UNKNOWNS; modelled matrix corrected for Type 3 recruits		Larval behaviour / Spawning regime combinations											
		PaS1	PaS2	PaS3	PaS4	OmS1	OmS2	OmS3	OmS4	BlS1	BlS2	BlS3	BlS4
Partitioning of the core region / Confidence level combinations	3x3%99	0.21	0.27	0.34	0.47	0.22	0.30	0.35	0.43	-0.13	-0.11	-0.09	-0.06
	3x3%95	0.43	0.50	0.56	0.68	0.43	0.50	0.56	0.62	0.05	0.07	0.09	0.13
	3x3%90	0.61	0.68	0.75	0.84	0.60	0.68	0.74	0.79	0.20	0.22	0.25	0.29
	3x3%75	0.79	0.85	0.91	**0.97**	0.76	0.83	0.88	0.90	0.42	0.44	0.48	0.51
	3x3%50	0.82	0.87	0.90	0.91	0.77	0.82	0.86	0.85	0.51	0.53	0.56	0.59
	3x3 Better	0.79	0.84	0.87	0.88	0.74	0.79	0.83	0.81	0.50	0.52	0.55	0.58
	3x4%99	0.31	0.35	0.40	0.49	0.28	0.33	0.38	0.43	-0.22	-0.21	-0.20	-0.18
	3x4%95	0.48	0.54	0.59	0.67	0.44	0.50	0.56	0.60	-0.04	-0.03	-0.01	0.02
	3x4%90	0.59	0.65	0.70	0.78	0.53	0.61	0.67	0.71	0.11	0.13	0.15	0.18
	3x4%75	0.67	0.74	0.77	0.84	0.62	0.70	0.76	0.78	0.33	0.35	0.37	0.40
	3x4%50	0.56	0.62	0.64	0.67	0.52	0.59	0.64	0.65	0.44	0.45	0.47	0.49
	3x4 Better	0.50	0.56	0.58	0.61	0.47	0.54	0.58	0.59	0.43	0.44	0.46	0.48
	4x4%99	0.42	0.46	0.50	0.56	0.38	0.42	0.46	0.50	-0.15	-0.13	-0.12	-0.10
	4x4%95	0.54	0.58	0.63	0.70	0.50	0.55	0.60	0.65	-0.05	-0.03	-0.02	0.00
	4x4%90	0.61	0.66	0.70	0.77	0.56	0.62	0.67	0.72	0.04	0.06	0.08	0.09
	4x4%75	0.69	0.74	0.78	0.84	0.64	0.71	0.76	0.79	0.23	0.24	0.26	0.26
	4x4%50	0.60	0.66	0.68	0.72	0.56	0.62	0.67	0.68	0.42	0.42	0.45	0.43
	4x4 Better	0.57	0.62	0.63	0.66	0.54	0.59	0.63	0.64	0.44	0.44	0.47	0.45

E

CORE MATRICES + UNKNOWNS modelled matrix corrected for Type 2 and Type 3 recruits		Larval behaviour / Spawning regime combinations											
		PaS1	PaS2	PaS3	PaS4	OmS1	OmS2	OmS3	OmS4	BIS1	BIS2	BIS3	BIS4
Partitioning of the core region / Confidence level combinations	3x3%99	0.47	0.55	0.61	0.76	0.51	0.58	0.62	0.71	0.07	0.09	0.11	0.14
	3x3%95	0.63	0.71	0.77	0.88	0.65	0.72	0.76	0.83	0.20	0.23	0.25	0.28
	3x3%90	0.69	0.77	0.83	0.93	0.71	0.78	0.83	0.88	0.26	0.29	0.32	0.35
	3x3%75	0.70	0.77	0.82	0.88	0.70	0.77	0.82	0.83	0.36	0.39	0.42	0.45
	3x3%50	0.57	0.63	0.66	0.66	0.55	0.61	0.65	0.63	0.35	0.37	0.39	0.41
	3x3 Better	0.53	0.58	0.62	0.61	0.50	0.56	0.60	0.57	0.33	0.35	0.38	0.40
	3x4%99	0.55	0.60	0.63	0.73	0.52	0.57	0.61	0.67	-0.06	-0.04	-0.03	-0.02
	3x4%95	0.63	0.70	0.74	0.83	0.60	0.66	0.71	0.76	0.08	0.10	0.11	0.13
	3x4%90	0.63	0.70	0.74	0.83	0.59	0.66	0.72	0.78	0.16	0.18	0.20	0.22
	3x4%75	0.56	0.63	0.67	0.73	0.54	0.61	0.67	0.70	0.28	0.30	0.31	0.33
	3x4%50	0.32	0.37	0.39	0.42	0.31	0.36	0.41	0.42	0.30	0.31	0.33	0.34
	3x4 Better	0.25	0.30	0.32	0.35	0.24	0.30	0.34	0.35	0.28	0.29	0.31	0.32
	4x4%99	0.89	0.90	0.90	0.92	0.84	0.85	0.86	0.86	0.47	0.46	0.48	0.43
	4x4%95	0.89	0.92	0.93	**0.96**	0.86	0.88	0.90	0.92	0.48	0.47	0.49	0.45
	4x4%90	0.86	0.90	0.91	0.95	0.83	0.86	0.89	0.91	0.47	0.46	0.49	0.45
	4x4%75	0.75	0.79	0.82	0.86	0.73	0.78	0.81	0.83	0.45	0.44	0.47	0.43
	4x4%50	0.33	0.38	0.41	0.46	0.32	0.38	0.43	0.45	0.22	0.23	0.25	0.25
	4x4 Better	0.21	0.26	0.29	0.34	0.21	0.27	0.32	0.34	0.14	0.15	0.17	0.17
