# Supplementary material for: Independent estimates of marine population connectivity are more concordant when accounting for uncertainties in larval origins
Source: Sci Rep. 2018 Feb 8;8:2641. doi: 10.1038/s41598-018-19833-w (PMC5805787; doi:10.1038/s41598-018-19833-w)
Supplement: Supplementary file 4 — Supplementary Information 4 [file 41598_2018_19833_MOESM4_ESM.pdf]

Supplementary Information 4: matrix adjustment

Independent estimates of marine population connectivity are more concordant when accounting for uncertainties in larval origins

Nolasco R[1,2], Gomes I[3,4], Peteiro L[3,5], Albuquerque R[3], Luna T[1], Dubert J[1], Swearer SE[6], Queiroga H[1]*

[1] Departamento de Física & CESAM - Centro de Estudos do Ambiente e do Mar, Universidade de Aveiro, 3810-193 Aveiro, Portugal

[2] Instituto de Investigacións Mariñas (CSIC), Eduardo Cabello 6, 36208 Vigo, Spain

[3] Departamento de Biologia & CESAM - Centro de Estudos do Ambiente e do Mar, Universidade de Aveiro, 3810-193 Aveiro, Portugal

[4] Marine Biology Research Group, Ghent University, 9000 Ghent, Belgium

[5] Coastal Ecology Research Group (EcoCost), Department of Ecology and Animal Biology, University of Vigo, Spain

[6] School of BioSciences, University of Melbourne, Parkville, Victoria, 3010, Australia

Corresponding author*

Henrique Queiroga: henrique.queiroga@ua.pt

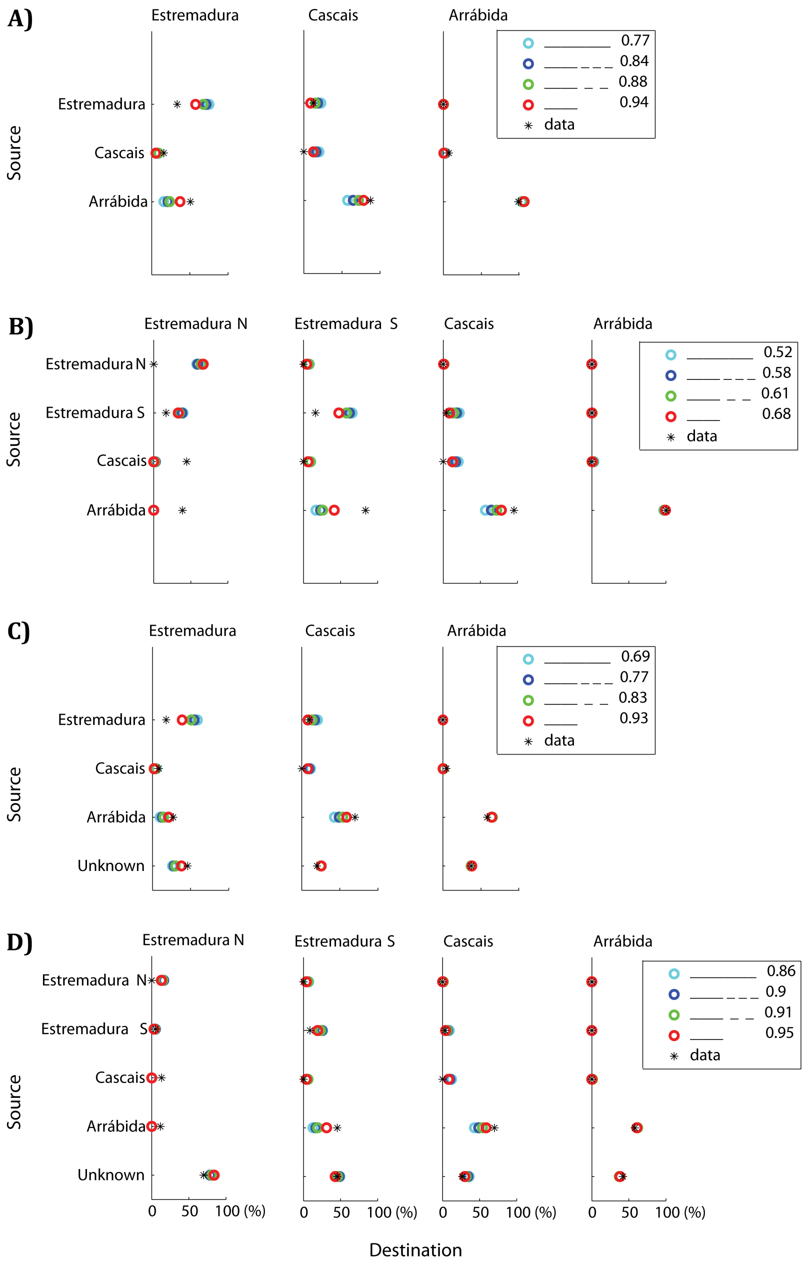

Supplementary Figure 4.1. Graphical comparison between Observed and Modelled connectivity matrices with two different spatial grid arrangements, for the case of passive behaviour, 90% confidence level and the four spawning regimes (colours). Data indicates observations. A) 3x3 core connectivity matrices uncorrected for Type 2 and/or Type 3 recruits; B) 4x4 core connectivity matrices uncorrected for Type 2 and/or Type 3 recruits; C) 3x3 core connectivity matrices corrected for Type 2 and Type 3 recruits plus unknown row; D) 4x4 core connectivity matrices corrected for Type 2 and Type 3 recruits plus unknown row.

Accounting for uncertainty increases the fit between observed and modelled connectivity matrices at higher spatial resolutions. In Supplementary Fig. 3.1 A) and B), the 3x3 spatial grid yields the best adjustment between the observed and modelled matrices. In Supplementary Fig. 3.1 C) and D), the 4x4 spatial grid yields the best correlations between the observed and modelled matrices. Accuracy greatly increases in the Estremadura regions for the corrected case.
