# Supplementary material for: Independent estimates of marine population connectivity are more concordant when accounting for uncertainties in larval origins
Source: Sci Rep. 2018 Feb 8;8:2641. doi: 10.1038/s41598-018-19833-w (PMC5805787; doi:10.1038/s41598-018-19833-w)
Supplement: Supplementary file 5 — Supplementary Information 5 [file 41598_2018_19833_MOESM5_ESM.pdf]

Supplementary Information 5: prediction 1

Independent estimates of marine population connectivity are more concordant when accounting for uncertainties in larval origins

Nolasco R[1,2], Gomes I[3,4], Peteiro L[3,5], Albuquerque R[3], Luna T[1], Dubert J[1], Swearer SE[6], Queiroga H[1*]

[1] Departamento de Física & CESAM - Centro de Estudos do Ambiente e do Mar, Universidade de Aveiro, 3810-193 Aveiro, Portugal
[2] Instituto de Investigacións Mariñas (CSIC), Eduardo Cabello 6, 36208 Vigo, Spain
[3] Departamento de Biologia & CESAM - Centro de Estudos do Ambiente e do Mar, Universidade de Aveiro, 3810-193 Aveiro, Portugal
[4] Marine Biology Research Group, Ghent University, 9000 Ghent, Belgium
[5] Coastal Ecology Research Group (EcoCost), Department of Ecology and Animal Biology, University of Vigo, Spain
[6] School of BioSciences, University of Melbourne, Parkville, Victoria, 3010, Australia

Corresponding author*
Henrique Queiroga: henrique.queiroga@ua.pt

A)

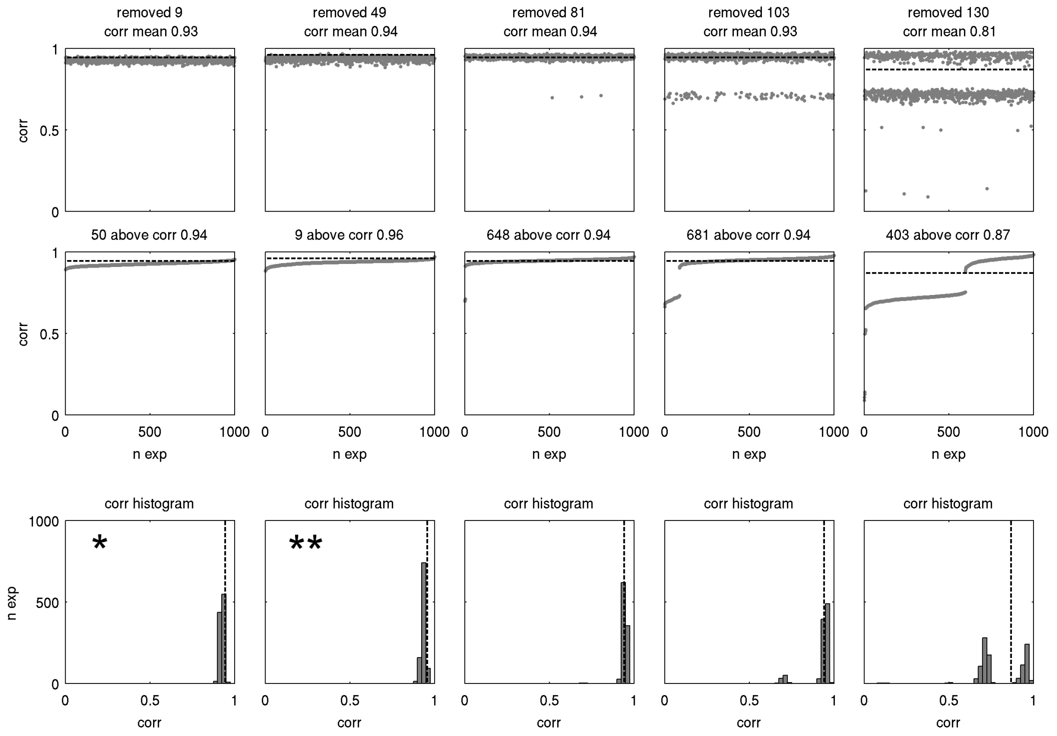
removed 9
corr mean 0.93
removed 49
corr mean 0.94
removed 81
corr mean 0.94
removed 103
corr mean 0.93
removed 130
corr mean 0.81
50 above corr 0.94
9 above corr 0.96
648 above corr 0.94
681 above corr 0.94
403 above corr 0.87
corr
n exp
corr histogram
*
**

B)

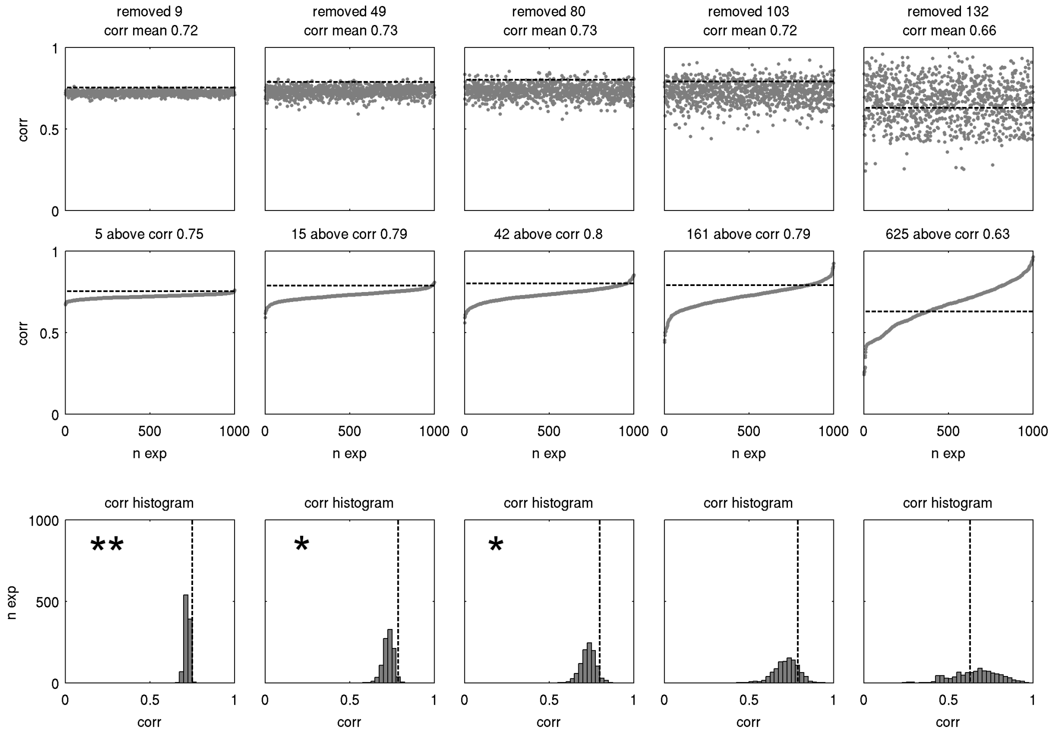
removed 9
corr mean 0.72
removed 49
corr mean 0.73
removed 80
corr mean 0.73
removed 103
corr mean 0.72
removed 132
corr mean 0.66
5 above corr 0.75
15 above corr 0.79
42 above corr 0.8
161 above corr 0.79
625 above corr 0.63
corr
n exp
corr histogram
**
*
*

C)

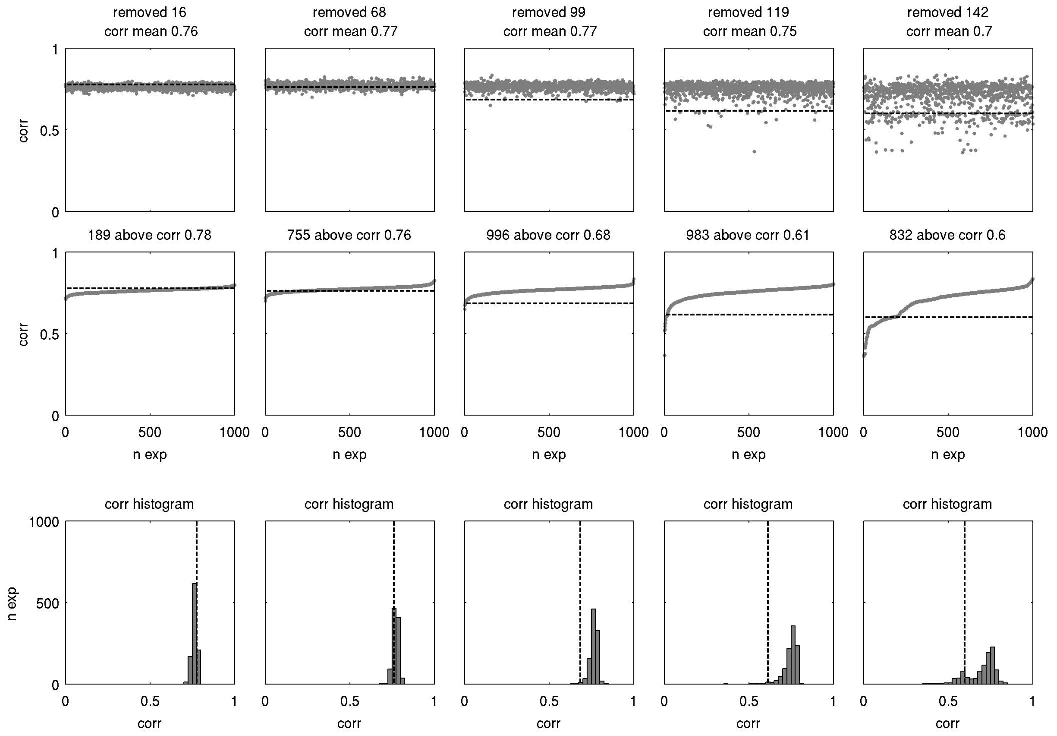
removed 16
corr mean 0.76
removed 68
corr mean 0.77
removed 99
corr mean 0.77
removed 119
corr mean 0.75
removed 142
corr mean 0.7
189 above corr 0.78
755 above corr 0.76
996 above corr 0.68
983 above corr 0.61
832 above corr 0.6
corr
n exp
corr histogram

D)

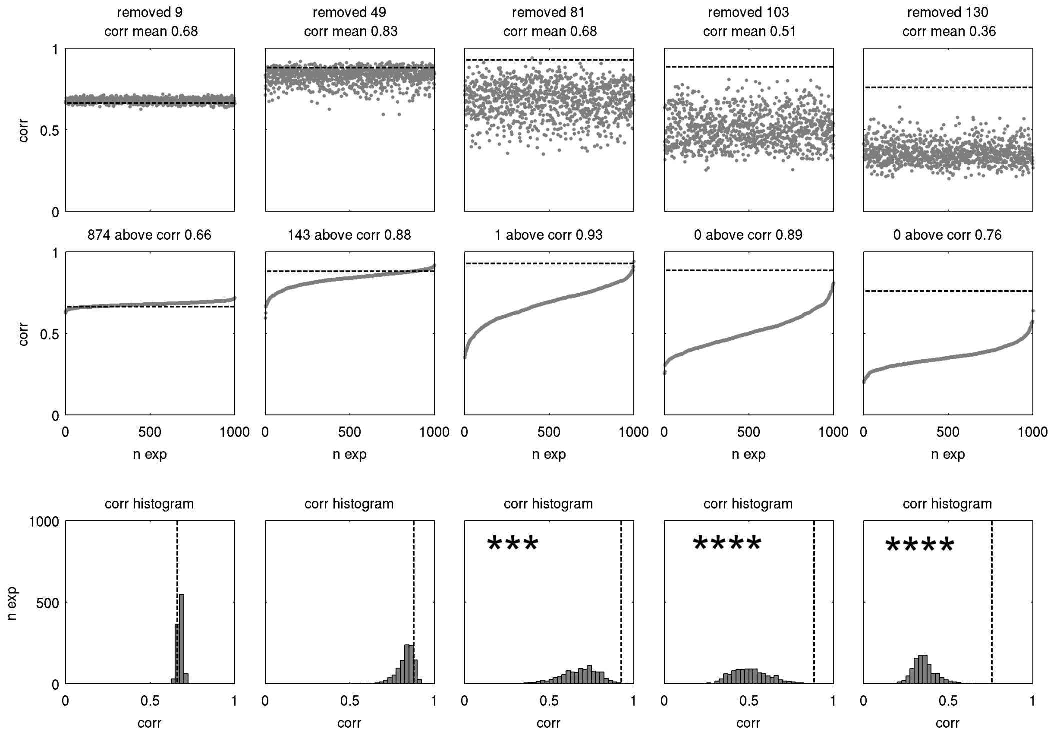
removed 9
corr mean 0.68
removed 49
corr mean 0.83
removed 81
corr mean 0.68
removed 103
corr mean 0.51
removed 130
corr mean 0.36
874 above corr 0.66
143 above corr 0.88
1 above corr 0.93
0 above corr 0.89
0 above corr 0.76
corr
n exp
corr histogram
***
****
****

E)

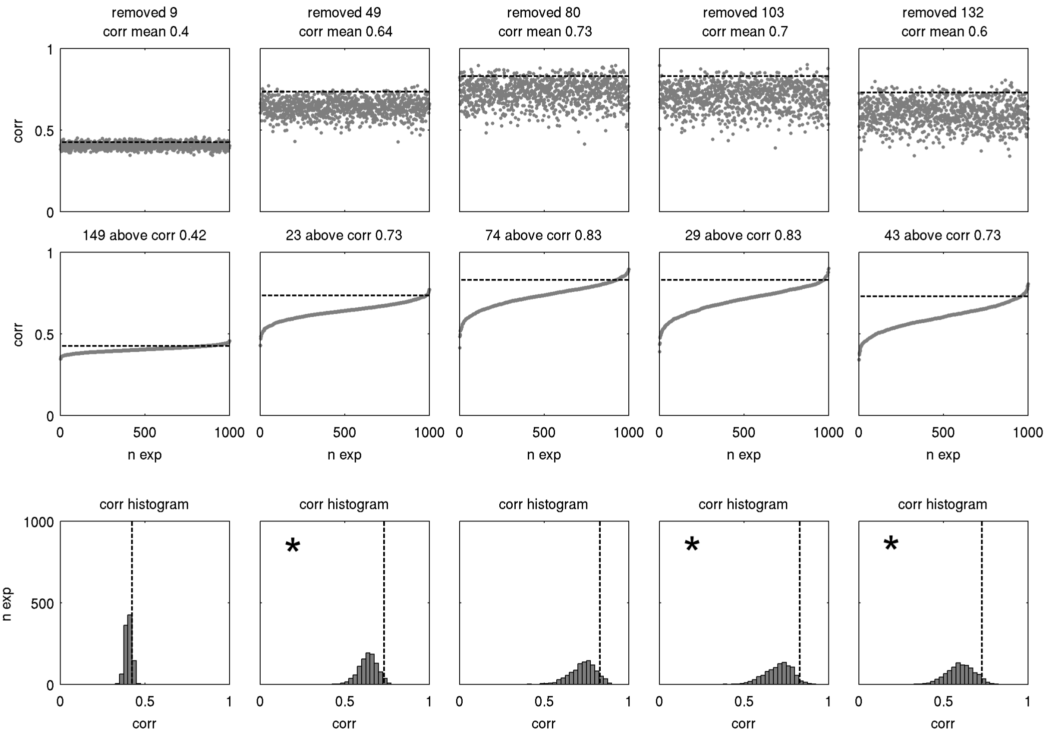

F)

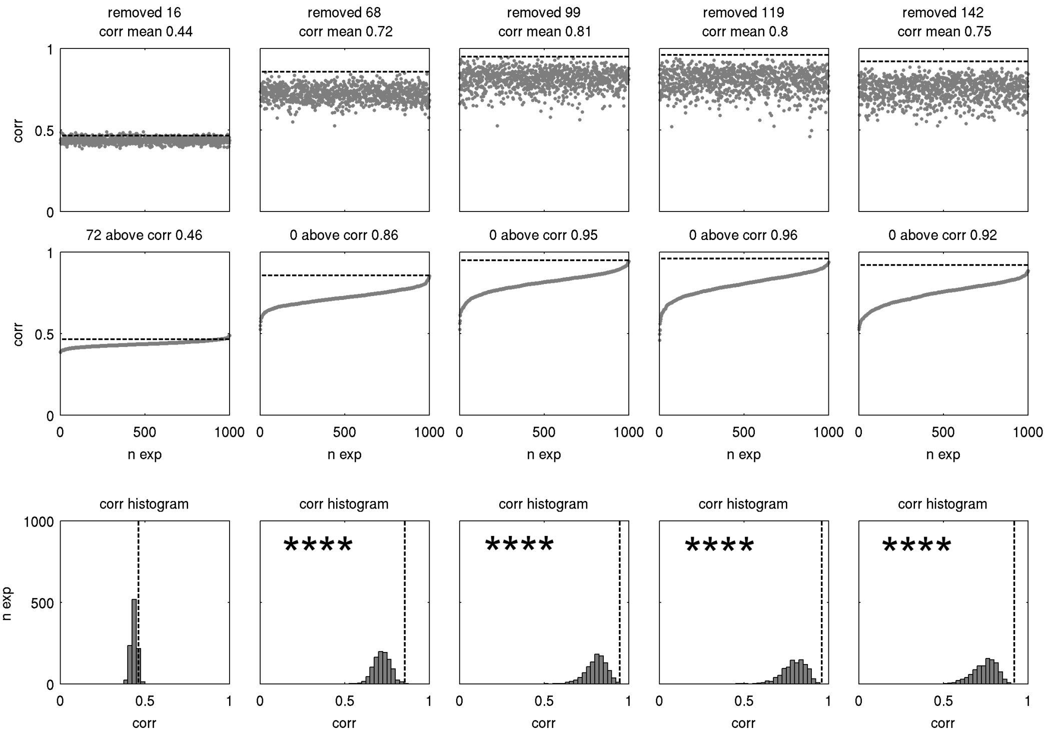

Supplementary Figure 5.1. Effect on the matrix correlation coefficient of randomly excluding from the observed matrix a number of recruits equal to the number of observed recruits

correctly classified as unknowns for each confidence level (columns are Assignment Probability Thresholds (APT) of 0.50, 0.75, 0.90, 0.95 and 0.99), based on 1000 trials for each threshold. Only the cases for continuous larval emission during each high tide until July 1 (S4) and passive larvae (Pa) were tested. A) 3x3 spatial arrangement, core connectivity matrices; B) 3x4 spatial arrangement, core connectivity matrices; C) 4x4 spatial arrangement, core connectivity matrices; D) 3x3 spatial arrangement, connectivity matrices with unknown row; E) 3x4 spatial arrangement, connectivity matrices with unknown row; F) 4x4 spatial arrangement, connectivity matrices with unknown row. In panels D) - F) the unknown row is composed of the recruits that were randomly assigned as unknowns in the case of the observed matrix, and of Type 2 and Type 3 recruits in the case of the modelled matrix.

In each graph, the dashed line indicates the correlation coefficient that was obtained by removing those recruits that correctly failed to pass the posterior probability threshold. First row of each panel: distribution of correlation coefficients ranked by trial number; the number of removed individuals is indicated above each graph. Second row of each panel: the same, but correlation coefficients ranked by value; the number of trials with a correlation coefficient above that obtained by removing those recruits that correctly failed to pass the posterior probability threshold is indicated above each graph. Third row of each panel: frequency distribution of the correlation coefficients. Removing the recruits that correctly failed to pass the APT resulted in a correlation coefficient significant higher than that obtained by a random deletion of recruits, at $p < 0.05$, 0.01, 0.001 and 0.0001 (*, **, ***, ****, respectively). "corr"= correlation coefficient.
