# Supplementary material for: Independent estimates of marine population connectivity are more concordant when accounting for uncertainties in larval origins
Source: Sci Rep. 2018 Feb 8;8:2641. doi: 10.1038/s41598-018-19833-w (PMC5805787; doi:10.1038/s41598-018-19833-w)
Supplement: Supplementary file 6 — Supplementary Information 6 [file 41598_2018_19833_MOESM6_ESM.pdf]

Supplementary Information 6: prediction 2

Independent estimates of marine population connectivity are more concordant when accounting for uncertainties in larval origins

Nolasco R[1,2], Gomes I[3,4], Peteiro L[3,5], Albuquerque R[3], Luna T[1], Dubert J[1], Swearer SE[6], Queiroga H[1*]

[1] Departamento de Física & CESAM - Centro de Estudos do Ambiente e do Mar, Universidade de Aveiro, 3810-193 Aveiro, Portugal
[2] Instituto de Investigacións Mariñas (CSIC), Eduardo Cabello 6, 36208 Vigo, Spain
[3] Departamento de Biologia & CESAM - Centro de Estudos do Ambiente e do Mar, Universidade de Aveiro, 3810-193 Aveiro, Portugal
[4] Marine Biology Research Group, Ghent University, 9000 Ghent, Belgium
[5] Coastal Ecology Research Group (EcoCost), Department of Ecology and Animal Biology, University of Vigo, Spain
[6] School of BioSciences, University of Melbourne, Parkville, Victoria, 3010, Australia

Corresponding author*
Henrique Queiroga: henrique.queiroga@ua.pt

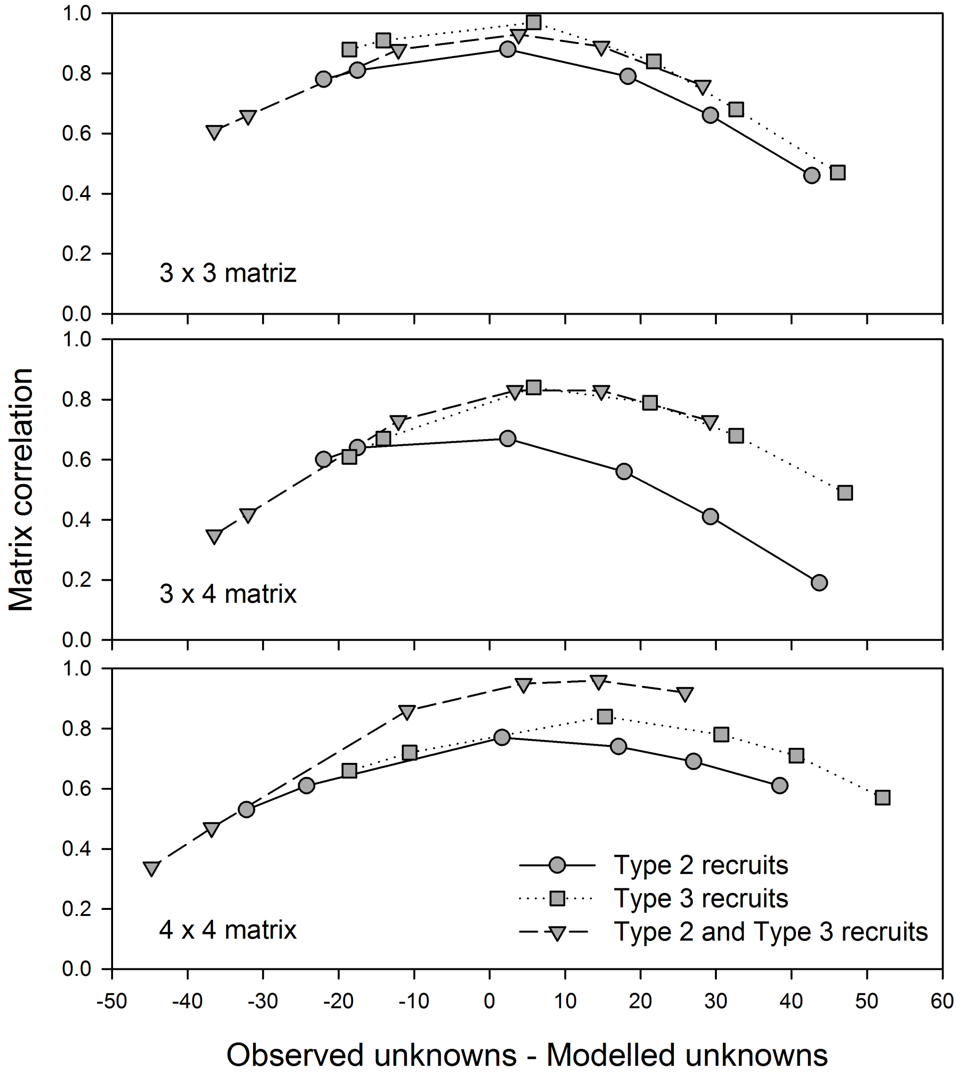

Supplementary Figure 6.1. Relationship between the matrix correlation coefficient and the difference between the numbers of observed and modelled recruits classified as unknowns, for three different arrangements of the connectivity matrices. The number of observed recruits classified as unknowns changes with the threshold level (from left to right, APTs better-than-the-rest, then 0.50, 0.75, 0.90, 0.95 and 0.99). The number of modelled recruits classified as unknowns depends on the misclassification rate of the larvae into their source population (proportion of larvae incorrectly self-assigned in each region; Type 2 recruits) and on those that originate from outside the core region (Type 3 recruits). The figure only shows results for passive larvae and the S4 spawning

scenario, separately for cases where only Type 2, only Type 3, and Type 2 and Type 3 recruits were classified as unknowns, for the same biological scenario.

The correlation coefficient (Supplementary Fig. 5.1) peaks at a difference close to zero, for an Assignment Probability Threshold (APT) of 0.75, when only Type 2 recruits are classified as unknowns, and at a differences between 0 and 20, for APTs of 0.90 and 0.95, when Type 2 and Type 3 recruits were classified as unknowns.
